# Navigating Body Perception, Affectivity, Intimacy, Gender Identity, and Sexuality: An Exploratory Qualitative Study in Young Adults with SMI, IDs, and ASD in a Community Setting

**DOI:** 10.3390/ijerph22050722

**Published:** 2025-05-01

**Authors:** Miriam Belluzzo, Veronica Giaquinto, Daniela Volpe, Camilla Esposito, Erica De Alfieri, Anna Lisa Amodeo

**Affiliations:** 1 Department of Mental, Physical Health and Preventive Medicine, University of Campania “Luigi Vanvitelli”, Largo Madonna delle Grazie 1, 80138 Napoli, Italy; erica.dealfieri@unicampania.it; 2 Department of Humanities, University of Naples “Federico II”, Porta di Massa 1, 80138 Napoli, Italy; veronica.giaquinto@unina.it (V.G.); daniela.volpe2@studenti.unina.it (D.V.); amodeo@unina.it (A.L.A.); 3 SInAPSi Centre, University of Naples “Federico II”, Via Giulio Cesare Cortese 29, 80138 Napoli, Italy; camilla.esposito@unina.it

**Keywords:** severe mental illness, intellectual disabilities, autism, sexuality, trauma-informed care, bodily autonomy, affectivity, young adults

## Abstract

This exploratory qualitative study investigates the challenges faced by young adults (aged 18–40) with severe mental illness (SMI), intellectual disabilities (IDs), and autism spectrum disorder (ASD) in navigating body perception, affectivity, intimacy, gender identity, and sexuality. Through semi-structured interviews with 13 participants recruited from a community habilitation center, thematic analysis revealed systemic barriers rooted in societal stigma, familial overprotection, and institutional neglect. Key findings highlight tensions between bodily autonomy and familial control, with prolonged caregiver dependency in intimate hygiene reflecting patterns of infantilization. We found that participants’ self-image was shaped by gendered expectations and familial critiques, reinforcing internalized stigma. Romantic relationships were often idealized through cultural narratives, yet lacked practical guidance on consent or boundaries, exacerbating vulnerabilities. Fragmented sexual education left individuals reliant on pornography or peers, perpetuating misconceptions and anxiety. Despite these barriers, participants demonstrated resilience and agency in advocating for inclusive interventions. This study underscores the urgent need for trauma-informed, rights-based approaches that prioritize autonomy, dignity, and intersectionality. Recommendations include structured skill-building programs for independence, disability-adapted comprehensive sexuality education, and systemic reforms fostering interdisciplinary collaboration. By centering lived experiences, this work challenges structural inequities and advocates for community care models that honor the diverse needs of individuals navigating the intersection of disability, identity, and sexuality.

## 1. Introduction

Sexuality, affectivity, intimacy, and gender identity construction are fundamental aspects of human experience, integral to overall well-being and quality of life. For individuals with severe mental illness (SMI), intellectual disability (ID) including genetic syndromes such as Down and Fragile X syndromes, and autism spectrum disorder (ASD), these dimensions of life are often overlooked or stigmatized, leading to significant challenges in achieving sexual and affective well-being [1,2,3,4]. Despite growing recognition of the importance of sexual health as a human right, as emphasized by the World Health Organization [5], individuals with these clinical conditions frequently face barriers to expressing their sexuality, forming intimate relationships, and exploring their gender identities [4,6,7]. This is compounded by societal misconceptions, inadequate sexual education, and a lack of supportive interventions tailored to their unique needs [4,5,7,8]. Critical gaps persist in understanding how these barriers operate across socio-ecological systems (micro, meso, macro) and intersect with clinical heterogeneity (e.g., co-occurring SMI, ID, ASD). Existing research often siloes these populations, neglecting shared psychosexual needs and the role of systematic oppression mechanisms in perpetuating exclusion.

Grounded in Isaac Prilleltensky’s critical community psychology framework [9,10,11,12] and Bronfenbrenner’s socio-ecological model [13,14,15], this study examines how power imbalances and structural violence manifest across micro-, meso-, and macro-systems to marginalize individuals with intellectual and mental disabilities. Prilleltensky’s [9,10,11,12] emphasis on well-being, oppression, and liberation provides a psychologically grounded lens to analyze: (1) how psychosexual marginalization results from interdependent systems of power (familial, institutional, and cultural); and (2) how participatory, rights-based interventions can foster emancipation. This dual framework addresses two key limitations in existing research: (1) the absence of integrated theoretical frameworks to examine and address sexual and affective marginalization faced by these populations across interconnected individual, familial, institutional, and cultural domains (e.g., efforts to develop effective sexual education programs will remain inadequate without simultaneously addressing structural dynamics tied to individual needs, familial contexts, and community norms); and (2) the underrepresentation of interventions for clinically diverse populations that are grounded in lived experiences and shared sexual and affective needs.

Research [16,17] has shown that individuals diagnosed with severe psychiatric illnesses, such as schizophrenia and other psychotic disorders, experience significant difficulties in forming and maintaining romantic relationships. These challenges stem from social isolation, cognitive deficits, and stigma, and are compounded by antipsychotic medications that often cause sexual dysfunction and reduced libido, significantly impairing their capacity to form and sustain intimate relationships [6,8,18].

On the other hand, individuals with intellectual disabilities, including those with genetic syndromes, often face infantilization and a denial of their sexual rights, leaving them vulnerable to exploitation, abuse, and risky sexual behaviors [4,18,19]. The lack of appropriate sexual education and support further compounds these risks as many individuals with intellectual disabilities struggle to understand social boundaries and reproductive health [20].

For autistic individuals, the intersection of autism with gender identity and sexual orientation presents additional complexities. Studies [7,21] have highlighted a higher prevalence of non-heterosexual orientations and gender dysphoria among autistic individuals, exposing them to dual stigma within both the autistic and LGBTQ+ communities. Autistic individuals also face unique challenges in navigating romantic and sexual relationships due to difficulties in social communication and sensory sensitivities, which can hinder their ability to form intimate connections and increase their vulnerability to victimization and abuse [22,23].

The risks faced by these populations are not merely theoretical: they have tangible consequences. Individuals diagnosed with severe psychiatric illnesses are at higher risk of engaging in risky sexual behaviors, such as unprotected sex and multiple sexual partners, which increase their vulnerability to sexually transmitted infections (STIs) and unintended pregnancies [6,24,25]. Similarly, individuals with intellectual disabilities and autism are more likely to experience sexual coercion and exploitation due to their difficulties in understanding social cues and asserting boundaries [26,27]. These risks are further compounded by the lack of comprehensive sexual education and supportive interventions, leaving these individuals ill-equipped to navigate the complexities of sexuality and relationships [4,28,29].

Despite the well-documented benefits of comprehensive sexuality education (CSE) for these populations [17,30,31,32], numerous practical challenges remain in its implementation. In educational, rehabilitation, and community settings, CSE programs often face constraints such as limited curriculum time, a shortage of trained educators and perceived parental opposition [28,33,34]. Moreover, financial limitations frequently hinder institutions from securing adequate technological and pedagogical resources, further impeding effective program development abilities [28]. In addition, it is particularly important to consider that in many rehabilitation and educational communities and settings, support services are provided under the umbrella terms of intellectual and mental disabilities, encompassing people with a diverse range of conditions—from psychosis to genetic syndromes like Down syndrome [35]—including those who present diagnoses that co-occur with more than one clinical condition. In fact, many studies [36,37,38] consistently report higher rates of psychotic disorders (e.g., schizophrenia) in individuals with an ID compared to the general population. Furthermore, certain genetic conditions (e.g., 22q11.2 deletion syndrome, Down and Fragile X syndromes) are linked to both IDs and an increased risk of psychosis [39,40,41,42,43]. Therefore, the clinical heterogeneity present in the educational and rehabilitation contexts devoted to those diagnosed with intellectual or mental disabilities collides with the lack of inclusive curricula suitable for a wide range of cognitive and psychiatric diagnoses and abilities [28,33,34,35]. Research [44,45] has shown that integrated intervention models—addressing shared needs—can be particularly effective in community settings where clients present with a wide range of conditions. This integrated perspective supports the development of interventions that are flexible, inclusive, and responsive to the complex realities of community care.

Addressing these challenges requires a multifaceted approach that recognizes the right of individuals with severe psychiatric illness, intellectual disability, and autism to sexual and affective well-being. This includes providing tailored sexual education, fostering inclusive environments that respect diverse gender identities and sexual orientations, and developing psychosexual interventions that address the unique needs of these populations [6,7] considering the limits of their social and educational contexts.

This exploratory study advances the growing body of knowledge by addressing three core objectives: (1) to investigate how young adults with SMI, ID, and ASD negotiate bodily autonomy, gender identity, and sexuality within their key ecological contexts, including familial, institutional, and rehabilitative settings; (2) to identify systemic barriers (e.g., stigma, inadequate sex education, infantilizing practices) that contribute to psychosexual marginalization across clinical sub-groups; and (3) to leverage participants’ lived experiences to inform rights-based interventions targeting shared psychosexual needs that are suitable for complex community and rehabilitation settings. Through the thematic analysis of semi-structured interviews with young adults (aged 18–40) diagnosed with SMI, ID, and ASD in a community-based rehabilitation setting, we map these intersections, exposing structural inequities that hinder inclusive approaches to disability and sexuality.

The term “young adulthood” lacks a universally accepted definition, often varying across research and clinical contexts. While psychological and sociological frameworks, such as Arnett’s [46] concept of “emerging adulthood”, typically focus on ages 18–25, this narrow scope fails to account for the prolonged and heterogeneous developmental trajectories of individuals with severe mental illness (SMI), intellectual disability (ID), and autism spectrum disorder (ASD). Developmental theorists like Erikson [47,48] conceptualize young adulthood as a phase extending into the fourth decade of life, during which milestones such as occupational stability, emotional regulation, and sexual identity consolidation unfold unevenly and are shaped by psychosocial, biological, and environmental factors [49]. For individuals with SMI, ID, and ASD, normative timelines of relationship formation and sexual exploration are often disrupted by persistent symptoms, diagnostic overshadowing, institutionalization, or delayed autonomy [6,7,50,51]. Adopting a broader age range (18–40 years) not only aligns with developmental theory but also reflects an ethical commitment to capturing underrepresented experiences and addressing systemic barriers to care. This inclusive approach enables a comprehensive exploration of how sexuality, intimacy, and gender identity evolve amid ongoing clinical challenges, whilst centering the diverse needs of individuals often excluded from typical definitions of young adulthood. To achieve this, the research objectives are operationalized through deliberate methodological choices: an exploratory qualitative design enables nuanced investigation of lived experiences, while purposeful sampling of a heterogeneous clinical population (spanning SMI, ID, and ASD) within a rehabilitation community setting ensures ecological validity. This approach facilitates a multilayered analysis of affectivity, sexuality, body perception, and gender identity that remains firmly grounded in participants’ sociocultural contexts.

The resulting insights provide a foundation for identifying cross-diagnostic psychosexual needs—a crucial step in developing tailored psychoeducational interventions that respect neurodevelopmental diversity while addressing common barriers. More than simply documenting challenges, this study’s participant-centered methodology actively amplifies marginalized voices, elucidating how young adults with severe psychiatric conditions, intellectual disabilities, and autism navigate structural constraints in their pursuit of sexual–affective well-being. Theoretical coherence is maintained through Prilleltensky’s [9,10,11,12] participatory ethos, used to challenge structural inequities through emancipatory research that bridges disability studies, clinical and community psychology, and sexual health advocacy. This study’s ultimate contribution lies in advancing the discourse on intervention paradigms that are simultaneously inclusive (addressing diverse clinical needs) and transformative (targeting systemic barriers). The findings advocate for rights-based approaches recognizing the inseparable interplay of biological, psychological, and social determinants of sexual health. By transcending reductionist diagnostic categorizations, this study affirms the need for multilevel solutions responsive to participants’ complex socioecological realities—a concrete application of Prilleltensky’s psycho-political validity principle [9,10,11,12].

## 2. Materials and Methods

### 2.1. Design of the Study and Participants

The present exploratory qualitative study investigates body perception, affectivity, sexuality, intimacy, and gender identity construction among young adults (aged 18–40) diagnosed with severe mental illness, intellectual disabilities (including genetic syndromes), and autism. Given the sensitivity and complexity of these topics, a qualitative design was selected to prioritize depth and nuance of participants’ lived experiences.

Qualitative inquiry is uniquely suited to under-researched phenomena, enabling hypothesis generation and thematic discovery [52,53]. Thematic analysis [54] was employed to systematically identify patterns across data, while a heterogeneous sample—spanning diagnostic groups—allowed for cross-condition comparisons of shared psychosexual and emotional needs. These insights aim to inform tailored psychosexual interventions for diverse clinical populations.

Participants were recruited from the Argo Center (FOQUS Foundation, Naples, Italy), a private multifunctional day habilitation facility serving individuals with severe mental illness (SMI), intellectual/developmental disabilities (IDDs), and autism. The center’s focus on fostering autonomy across life domains (e.g., education, employment, psychosocial well-being) aligned with this study’s goal of addressing systemic barriers to sexual and affective health.

#### 2.1.1. Ethics

Informed consent was obtained from service users of the Argo Center who were able to understand it and express their willingness to take part in this study. Additionally, as an extra precaution, authorization was also sought from their parents or legal guardians, who signed an informed consent form for their children or dependents to participate in this study. This additional step was deemed necessary given the sensitive nature of the topic being addressed. The research team felt it was important that parents were fully informed about the project and its objectives, and that they approved and consented to the participation of their children or dependents.

This study followed the guidelines of the Declaration of Helsinki and was approved by the Ethical Committee of Psychological Research of the Department of Humanities of the University of Naples ‘Federico II’ (protocol no. 13/2024).

#### 2.1.2. Participants

##### Inclusion Criteria

Eligible participants for the pilot study were recruited considering the following criteria: (1) verified diagnosis of intellectual disability (including genetic syndromes), autism, and/or severe mental illness according to the criteria of the ICD-10 [55], Diagnostic and Statistical Manual of Mental Disorders, 5th edition [56], DSM-IV-TR [57]; (2) receiving services related to such disability; (3) aged between 18 and 40 years; (4) provided personal consent to participate in this study; (5) consent provided by parents or a legal guardian; (6) sufficient verbal and textual comprehension skills to understand the informed consent and to participate in the training.

Of the 40 potential participants initially identified by the Argo Center, 27 were excluded due to unmet inclusion criteria (e.g., inability to provide assent, insufficient comprehension skills, or lack of guardian consent). The final sample comprised 13 participants. While this modest sample size precludes statistical generalizability, qualitative research prioritizes rich, contextually grounded insights over numerical breadth [54]. The heterogeneity of the sample—spanning individuals with severe mental illness (SMI), intellectual/developmental disabilities (IDDs), and autism—ensured diverse perspectives on shared psychosocial needs, aligning with this study’s exploratory aims.

Recruiting individuals with severe psychiatric and developmental conditions necessitated balancing ethical safeguards (e.g., assent, guardian consent) with research goals. The stringent inclusion criteria, while reducing the sample size, ensured participants could engage meaningfully in interviews, thereby enhancing the credibility of their narratives [53]. The participants’ ability to contribute meaningfully underscores their capacity to shape research on deeply personal topics [7].

As an exploratory study, the primary objective was not to achieve saturation but to identify preliminary themes and hypotheses for future research. The findings thus provide a foundational understanding of under-researched experiences in a population often excluded from the discourse on sexuality and affectivity.

Socio-demographic characteristics of the participants are presented in Table 1. Each participant is presented with a code (F for female participants; M for male participants) in order to preserve their anonymity. For this study, no standard selection procedures were followed, and the identified target group can be considered a criterion-based convenience sample.

##### Exclusion Criteria

Exclusion criteria included (1) aged under 18 years old; (2) personal consent to participate in this study not provided; (3) parent or a legal guardian consent to participate in this study not provided; (4) not possessing sufficient verbal and textual comprehension skills to understand the informed consent and to participate in the training.

Participants were not excluded based on gender, race, ethnicity, sexual orientation, educational level, religion, and/or socioeconomic status.

#### 2.1.3. Procedures and Materials

As a preliminary step, Centro Argo convened a meeting in the third week of March 2024 with the parents and/or legal guardians of eligible individuals for the exploratory study. The meeting aimed to introduce the project and the research team, clarify its objectives, and distribute informed consent forms. Similarly, during the same week, another meeting was held with the services users who met the inclusion criteria, during which the research team presented the project, explained its voluntary nature, and read aloud the informed consent form, reformulating it where necessary to ensure comprehension, discussed its contents, and collected signed consent forms from those who expressed interest in participating.

Both meetings took place in the presence of the scientific director of Centro Argo, the coordinator of the educators’ team, and the educators scheduled for those days.

Before starting the interviews, the research team took part as participant observers in the activities of the Argo Center from March 2024 to the end of April 2024, with the aim of creating a transferal bond with the center’s users participating in the exploratory study and fostering the development of a climate of mutual trust and respect. This approach aligns with established qualitative research practices that emphasize the importance of building rapport and trust with participants, particularly when working with vulnerable populations or in sensitive contexts [58,59]. Participant observation allowed the research team to immerse themselves in the daily routines and social dynamics of the Argo Center, gaining a deeper understanding of the context in which participants lived and interacted. This immersion helped to reduce power imbalances between researchers and participants, creating a more equitable and respectful research environment [60,61]. By engaging in the activities of the Center, the research team were able to demonstrate their commitment to understanding the participants’ experiences, which facilitated the development of trust and openness during the subsequent interviews [58,59].

All the interviews were conducted by the first author (M.B.), who was assisted by the second (V.G.) and the third (D.V.) authors acting as observers. All the participants were interview individually in a private room provided by the Argo Center and separate from the common areas. The presence of observers allowed for a more comprehensive data collection process, as they took detailed notes on non-verbal cues, tone, and context, while the primary interviewer focused on guiding the conversation. This collaborative approach ensured that all key topics were covered and provided multiple perspectives during the analysis phase, enhancing the reliability and depth of the findings [58,59]. Participants were informed of the observer’s roles at the beginning of each interview, and their consent was obtained to ensure their comfort and willingness to proceed [59,62].

Given the cognitive and clinical vulnerabilities of the participants, the interviews were designed to be semi-structured, with significant flexibility to accommodate individual needs and preferences. Participants were encouraged to express themselves freely, guiding the conversation toward topics they found most relevant to their experiences. To ensure participants’ comprehension, a simplified language approach was employed and contents were adjusted as needed to meet individual requirements and preferences [63]. This participant-centered approach empowered individuals to share their stories in a safe and comfortable manner [58,59].

The semi-structured interviews explored five key areas: body perception, affectivity and emotional expression, intimate relationships, gender identity construction, and sexuality. These themes were selected to capture the multifaceted nature of affectivity, sexuality, and gender identity, while providing a structured yet adaptable framework for participants to share their experiences [58,59]. The semi-structured format ensured that key themes were consistently addressed across interviews, whilst allowing the participants to prioritize topics most meaningful to them [54,59]. The interview guide is provided in Appendix A, Table A1, and consists of open-ended questions (e.g., “What is the difference between the body you had as a child and the one you have now?”). Follow-up prompts (e.g., “Can you tell me more about that experience?”) were used to elicit more detailed responses.

To further reduce potential stress or fatigue, participants were given the option to split the interview into multiple sessions if they felt overwhelmed or needed more time to reflect on their responses. This flexibility was particularly important given the sensitive nature of the topics discussed and the potential emotional or cognitive strain that prolonged interviews might cause [62,64]. By allowing participants to decide how and when they wanted to share their experiences, we aimed to create a supportive and non-intrusive environment that prioritized their well-being [58,59].

### 2.2. Measures

Demographic information—including gender, year of birth, mental health diagnosis, and educational qualifications—was collected from existing records at the Argo Center.

All interviews were audio-recorded and subsequently transcribed verbatim in order to analyze the data thematically following the guidelines by Braun and Clarke [54].

#### Qualitative Analysis

**Figure 1:**
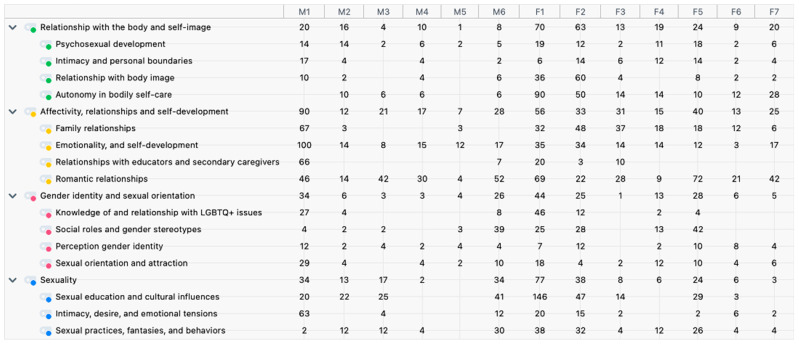
Summary table generated with MAXQDA24 of themes and sub-themes across interviews.

Thematic analysis followed Braun and Clarke’s six-phase framework [54], with MAXQDA24 software employed to facilitate collaborative coding, thematic mapping, and the systematic extraction and organization of data segments. To ensure analytical rigor and minimize bias, all phases involved iterative discussions among four researchers (M.B., V.G., D.V., and A.L.A.), with explicit protocols for resolving discrepancies. The phases included:

1. Familiarization: The research team (M.B., V.G. and D.V.) immersed themselves in the dataset by repeatedly reading interview transcripts and listening to audio recordings. Observational notes from participant engagement at the Argo Center were cross-referenced during weekly team meetings to contextualize narratives and identify preliminary patterns.
2. Initial Coding: Using MAXQDA24, two researchers (M.B. and V.G.) independently coded transcripts to capture semantic (explicit) and latent (underlying) meanings. An inductive approach ensured themes emerged organically. Coding disagreements (e.g., differing interpretations of emotional expression narratives) were flagged and discussed in biweekly debriefing sessions with the full team (M.B., V.G., D.V., and A.L.A.), leading to consensus through deliberative dialog. Codes were iteratively refined (e.g., merging overlaps, splitting broad codes) based on collective input.
3. Theme Development: Preliminary themes and sub-themes were collaboratively grouped during three dedicated workshops. MAXQDA24’s visualization tools (e.g., code matrices, thematic maps) enabled the team to compare interpretations and identify patterns across interviews. Discrepancies in thematic grouping (e.g., whether “body autonomy” should merge with “familial control”) were resolved through discussions, with final decisions arbitrated by A.L.A. to ensure coherence and enhance the analytical rigor.
4. Theme Review: Themes were critically evaluated against the full dataset by three researchers (M.B., V.G., and A.L.A.). This process involved re-examining raw transcripts to verify coherence, discarding themes that lacked empirical support (e.g., “sexual fantasies” was initially a standalone theme but merged into “sexual practices, fantasies and behaviors” after review), and reorganizing sub-themes to better reflect participants’ lived experiences. A.L.A. conducted an external audit of the thematic structure, challenging interpretations to strengthen validity. MAXQDA24 generated a summary table (Figure 1) to visually represent the definitive distribution, frequency, and hierarchical relationships of finalized themes and sub-themes across interviews. This table provided a clear overview of theme prevalence, aiding in the selection of the most relevant segments for in-depth analysis.
5. Defining Themes: Each theme was precisely labeled and defined through collaborative writing sessions led by the primary authors (M.B. and V.G.), supported by illustrative quotes. Analytical narratives were drafted by M.B. and V.G., then cross-reviewed by D.V. and A.L.A. to ensure interpretations aligned with the dataset and addressed potential biases and the research questions, emphasizing intersections between body perception, affectivity, and gender identity.
6. Reporting: Findings were synthesized into a structured narrative by M.B. and V.G., integrating participants’ voices to foreground their perspectives. MAXQDA24 facilitated the extraction and organization of representative quotes, ensuring alignment between the analysis and the final report. Representative quotes were selected collectively to ensure they authentically reflected the dataset.

To ensure reliability, M.B. and V.G. maintained reflexive memos in MAXQDA24 to document coding decisions, biases, and interpretive shifts. Regular debriefings minimized drift, while third-researcher triangulation (A.L.A.) strengthened validity. This iterative, team-based approach balanced depth and consistency, aligning with qualitative research best practices [45,47].

## 3. Results

Thematic analysis of the interviews allowed for an in-depth exploration of the experiences, perceptions, and relational dynamics of the participants diagnosed with ID, SMI and autism in relation to body management, affective development, gender identity, and sexuality. Through a systematic coding of the data, four main themes emerged, each structured into sub-themes that reflect the complexities of these dimensions.

The identified themes include: (1) relationship with the body and self-image, which examines autonomy in self-care, body image perception, and respect for personal boundaries; (2) affectivity, relationships, and self-development, which explores the role of family, romantic, and educational relationships in shaping identity; (3) gender identity and sexual orientation, which focuses on awareness of one’s gender, social influences on gender roles, and the understanding of sexual orientation; (4) sexuality, which delves into sexual behaviors, intimacy, and sex education, exploring individual sexual dynamics and cultural and familial influences. The analysis places particular emphasis on the emotional and psychological aspects of sexuality, including experiences, fears, desires, and the role of sex education, as well as the influence of pornography and the media.

These themes represent recurring patterns emerging from the data, offering a structured perspective on the challenges, opportunities, and influences shaping participants’ lived experiences. The analysis is supported by significant excerpts from the interviews, providing a direct and authentic illustration of participants’ personal experiences.

To facilitate a clear overview of the key findings, a summary table outlining the main themes and sub-themes is provided below (Table 2).

### 3.1. Theme 1: Relationship with the Body and Self-Image

The way participants diagnosed with IDs, SMI, and ASD perceive and manage their bodies is influenced by familial, social, and personal dynamics. Autonomy in self-care serves as a key indicator of development; however, a tendency towards infantilization and prolonged dependence on caregivers is evident. Body image is frequently shaped by social expectations and familial influences, resulting in both acceptance and discomfort. Additionally, respect for personal boundaries and intimacy are crucial elements that vary significantly among participants, with some demonstrating awareness and others exhibiting limited control over their personal space. The main sub-themes that emerged from the analysis include: (1) autonomy in bodily self-care; (2) relationship with body image; (3) intimacy and personal boundaries; (4) psychosexual development.

#### 3.1.1. Sub-Theme 1: Autonomy in Bodily Self-Care

The acquisition of autonomy in bodily self-care emerges as a heterogeneous process among the participants, reflecting varying degrees of independence. Some individuals, such as F5 and M3, demonstrate self-sufficiency in managing aspects of personal hygiene, confidently affirming their ability to handle menstruation or perform daily hygiene tasks independently:

I: When you get your period, who puts the pad on you?

F5 confidently responds: “Me.” (F5, Pos 194–195).

I: M3, do you wash by yourself?

M3: Of course, yes, I am on my own. (M3, Pos 73–74).

However, for others, parental involvement remains central, extending into adolescence and adulthood, often beyond the typical developmental trajectory of autonomy.

A recurring theme is the persistence of parental supervision in personal hygiene and bodily care, particularly among female participants. For example, F4 reports that her mother continues to bathe her, while F3 still relies on maternal assistance for managing her menstrual hygiene:

I: So, if I understand correctly, your mum showers you?

F4: Yes, sure! (F4, Pos 273–274).

I: When do you have your menstrual cycle? Your period… how do you manage it?

F3: Well, I wash.

I: Do you wash… alone or do you let mummy help you?

F3: Well, mummy puts the pad on me…now it’s not coming…I’m taking the painkiller.

I: Mmm… but do you know how to put the pad?

F3 makes a sound with her mouth indicating no. (F3, Pos 200–205).

This prolonged dependence highlights the intersection between autonomy, familial caregiving, and perceived vulnerability.

Another significant aspect concerns the involvement of opposite-sex caregivers in intimate hygiene practices. Cases such as F7, assisted by her father in washing, or F1, who received guidance from her father during showers until the age of 16, underscore dynamics that differ from the more common practice of same-sex parental support:

I: So, if I understand correctly, you wash by yourself, take a shower by yourself, do the bidet by yourself?

F7: Sometimes yes and sometimes no.

I: And who helps you with the bidet?

F7: Dad. (F7, Pos 109–112)

I: How did it work until you were 16?

F1: OK. Dad told me “Get everything off from head to toe’ and let’s start showering first”.

I: Was Daddy inside the same room or did he tell you from outside?

F1: In the same room. (F1, Pos 201–204)

Similarly, M2 reported relying on his mother’s assistance with personal hygiene until adulthood:

I: So, at 18 you started to do everything yourself and before that who helped you with personal hygiene?

M2: My mother. (M2, Pos 33)

These accounts suggest that familial care structures may override conventional social norms regarding gendered caregiving, prioritizing practical over normative considerations.

Despite these variations, the role of the family remains pivotal in shaping bodily autonomy. Parents act as primary facilitators of self-care education, guiding their children in learning to manage personal hygiene. Instances like M3, who attributes his acquired autonomy in washing and dressing to paternal instruction, or F1, who learned menstrual management from her mother, illustrate how familial guidance supports—though sometimes delays—the transition toward self-sufficiency or influences the use of inconvenient and outdated sanitary products:

I: And who taught you to wear pads, girdles?

F1: So, the pads my mother taught me how to put on. The girdle also my mother, then I always put it on myself as I want to and then I also learnt how to understand how to put on the girdle properly to cover the panties. (F1, Pos 235–236)

I: So, if I understand correctly, Dad taught you how to wash and dress yourself?

M3: Of course. (M3, Pos 115–116)

However, in certain cases, the persistence of parental involvement beyond the expected developmental timeline suggests potential overprotection, reinforcing dependency rather than fostering autonomy. F3, for example, continues to rely on her mother for personal hygiene:

I: But tell me something, F3…do you shower by yourself?

F3: No, my mum.

I: Does your mum help you?

F3: Oh yes, yes.

I: And why does your mum help you?

F3: I don’t know how to do it. (F3, Pos 192–197)

Ultimately, the findings indicate that while some participants achieve a degree of bodily autonomy, others experience a prolonged reliance on parental support, often shaped by familial perceptions of competence and vulnerability. These dynamics underscore the need for structured, gradual interventions that empower individuals to develop personal autonomy while respecting their pace of learning and adaptation.

#### 3.1.2. Sub-Theme 2: Relationship with Body Image

Body image emerges as a significant aspect of individual experience, influenced by external models and familial expectations. Clothing, hairstyle, and body hair management are key elements through which participants express their relationship with their bodies—sometimes asserting personal preferences, other times submitting to external decisions.

The aspiration to emulate a specific aesthetic model is evident in M1, who constructs his style by selectively adopting elements from a public figure: “I always copy A. [Italian singer]—the jackets, not the socks, the jackets, the ties. Exactly. Like, I don’t copy the shoes from A. I only copy one thing from A.: the glasses, the hats, the ties, the clarinets, the guitars, like the vests.” (M1, Pos 465). Here, clothing becomes a tool for identity construction and self-expression.

Physical changes also shape self-perception, as seen in M4 who associates hair loss with aging:

M4: I’m young, but a little bit old.

I: A little old already?

M4: I don’t have hair here anymore.

I: Ah, because you lost hair?

M4: I’m losing it.

I: So, for this reason, you feel old.

M4: Of course, yes. (M4, Pos 205–212)

A recurring theme is the tension between personal desires and family-imposed choices regarding appearance. F2 describes a hairstyle imposed by her parents that contradicts her personal preference:

I: Why do you have short hair?

F2: Because mom and dad make me cut it like a boy, since I was born.

I: Why?

F2: Because they don’t want long hair, that’s why! But my sister and I want long hair, not short! (F2, Pos 114–117)

Similarly, body hair management emerges as a crucial aspect of self-image. F2 expresses discomfort regarding body hair and describes the strategies she employs to remove it:

I: Do your body hairs bother you, or were they fine as they were?

F2: They bother me. I have them under my armpits, and at home, I wax my armpits and also my chest because I have hair there. Yes, on my chest, I remove them with a razor—here, here, and here with the razor. And here and here, I go to the beautician because I can’t do it myself. (F2, Pos 122–123).

The acceptance or rejection of one’s body varies among participants, with expressions of pride, dissatisfaction, or concern over specific physical features. M6, for instance, expresses confidence in his physique:

I: So, is M6 a boy? But is he a child or an adult?

M6: A boy with a great physique.

I: With a great physique…

M6: I’m in peak shape! (M6, Pos 90–93)

Conversely, F1 expresses concerns about breast size, associating it with weight gain and an undesired body aesthetic. The breast becomes a point of comparison with female relatives and former classmates and is linked to weight control: “But I don’t want to have gigantic breasts like my high school friends, my mom, or my sister. Because to have breasts like mine, you need to go on a diet, and when your breasts get too big, it means you’ve gained weight, and the more they grow, the more it means you’ve gained.” (F1, Pos 769).

Makeup use also plays a role in body image perception. F2 conveys a balanced approach, feeling beautiful both with and without makeup:

I: And how do you see yourself when you put on makeup?

F2: Eh, beautiful!

I: Do you think you’re more beautiful with or without makeup?

F2: Both. (F2, Pos 289–292)

Family influence on body image construction is evident through comments, aesthetic expectations, and choices not always shared by the participants. Parental pressure to conform to specific beauty standards is apparent in F1’s experience:

I: So, how did you start waxing? Did your mom ask you, or did you ask her?

F1: My mom asked me, because my mom always looks at my legs and says that I have too much hair, I need to go get waxed […].

I: So, is it more of your mom’s concern?

F1: Mom’s? Yes, also dad’s. He checked and told me, “F1, you’re hairy, go get a wax”. (F1, Pos 118–123)

Parental control extends beyond body hair, shaping attitudes toward makeup as well. F1 recalls how family regulations dictated her ability to wear cosmetics: “When I started wearing makeup at seven, at my cousin’s friends’ house… my mom said, ‘No, don’t let my daughter wear makeup, because I’m afraid she won’t know how to handle lipstick, makeup, and she’s not used to it.’ Then, when she saw me older, she let me put some makeup on my eyes. But when I put on lipstick by myself, she got mad and said, ‘Who told you to put on lipstick now that you’ve grown up?’ And I said, ‘I did, Dad…,’ and Dad told me, ‘No, F1, wipe it off’” (F1, Pos 308).

Furthermore, parental comments significantly impact self-perception. F3 internalizes a derogatory remark made by her mother:

I: (…) But who told you that you’re a whale?

F3: Mom.

I: And what do you say when she tells you that you are a whale?

F3: I get angry. (F3, Pos 160–163)

These narratives highlight how personal and familial dynamics shape body image, illustrating a complex interplay between individual identity, external influences, and internalized standards.

#### 3.1.3. Sub-Theme 3: Intimacy and Personal Boundaries

The theme of intimacy and personal boundaries emerges prominently in the narratives of the interviewees, revealing complex dynamics related to privacy, personal space, and autonomy. Participants demonstrate varying degrees of awareness and assertion of personal boundaries, alongside challenges in maintaining them within family and institutional settings. Several participants explicitly articulate discomfort when their physical space is invaded. For instance, M1 tolerates affectionate gestures but strongly resists unsolicited physical contact, stating, “No, no, but if someone caresses me, I don’t mind. But if someone touches me, I get angry like a beast” (M1, Pos 1492). This awareness extends to interactions with professionals, as M1 criticizes an inappropriate question posed by a therapist, underscoring the violation of his perception of bodily integrity: “For example, he [the therapist] says, ‘How’s your butt and your pee-pee?’ Do you think that’s normal for a 26-year-old?” (M1, Pos 531). Such responses highlight the importance of professionals maintaining appropriate boundaries in their communication with young adults.

Within the family context, experiences of personal space vary significantly. F5 describes a prolonged lack of a private sleeping space, having shared a bed with her father until the age of 16, and reports limited privacy in bathroom use, where family members habitually leave the door open despite her preference for closure: “I close it… but then they leave it open.” (F5, Pos 175). In contrast, F6 describes having a private room since childhood, demonstrating a greater degree of autonomy: “I have my own room… I sleep alone” (F6, Pos 19). Similarly, M1 and M4 confirm possessing their own rooms, indicating family recognition of their need for personal space. Conversely, F4 details a constrained living arrangement where she shares a bed with her mother due to space limitations: “Dad sleeps on the couch, Mom is with me… There’s no space” (F4, Pos 92).

The ability to assert and maintain boundaries is a crucial aspect of personal development. F1 describes a significant moment of self-determination when she insisted on showering independently: “I decided on my own to shower alone. Because I’m grown up. I’m not a little girl anymore” (F1, Pos 216). This statement reflects a shift towards self-awareness and the assertion of bodily autonomy. However, some participants exhibit uncertainty regarding boundaries, as seen in F4’s difficulty recalling whether she initiated or received a kiss:

I: Who kissed whom—G. kissed you, or you kissed G.?

F4: He kissed me… I kissed him. Dunno… (F4, Pos 559–560)

Such ambiguity suggests challenges in distinguishing personal agency from external actions.

For some participants, the concept of personal boundaries remains fluid within the family environment. F2, for example, describes instances of entering the bathroom while her brother showers, reinforcing a lack of firmly established privacy norms:

I: Does it happen that you go in while your brother showers?”

F2: It happens, it happens. (F2, Pos 206–207)

Similarly, she notes that her brother sometimes leaves the bathroom door open, reflecting inconsistent privacy practices: “Sometimes he leaves it open, sometimes he closes it” (F2, Pos 213).

These accounts illustrate diverse experiences in navigating intimacy and personal boundaries. While some participants assert their need for personal space and privacy, others struggle with blurred or poorly defined boundaries, particularly within family settings. These findings underscore the importance of fostering greater autonomy and respect for individual boundaries, both in family dynamics and professional interactions.

#### 3.1.4. Sub-Theme 4: Psychosexual Development

Psychosexual development presents a complex experience, often marked by a limited awareness and understanding of physical and sexual changes. When participants were asked about the difference between their childhood and current bodies, responses varied from vague acknowledgment to confusion. One participant mentioned a slight awareness (“A little bit there is” M2, Pos 40), while another showed uncertainty, asking, “What is the difference? I don’t know…” (M6, Pos 81). This lack of clarity reflects difficulties in recognizing specific bodily changes associated with growth and maturity. Additionally, when asked about the transition from childhood to adulthood, one participant answered:

F4: Two weeks ago.

I: (…) And what were you before?

F4: A female.

I: And now?

F4: I’m tall. (F4, Pos 316–322).

Suggesting a disconnection from the more profound social and physical transformations that occur.

Awareness of gender differences varied among participants. Some demonstrated clear recognition of anatomical distinctions, such as the presence of male and female genitalia (e.g., “There’s the penis”—F1 Pos 941; and M2 “You can tell from the… from the body that there’s… Let’s say a man’s body is there… let’s say the penis… The girl has breasts.”—M2 Pos 81), while others displayed more limited or less articulate understandings. For instance, one participant’s answer was simply to identify her gender, “Female” (F6, Pos 73), and when asked to define what it meant to be female, she responded, “Little girl” (F6, Pos 75), reflecting a perception of gender that remains anchored to childhood, suggesting either a difficulty in conceptualizing adulthood as distinct from childhood or an understanding of femininity primarily through an age-related framework. On the other hand, some participants, like F2, identified physical and gender-based distinctions more comprehensively, mentioning changes such as the growth of breasts, menstruation, or the development of facial hair in men.

The body’s sexual nature emerged more clearly in the context of sexuality and intimacy. Some participants, such as M2 and F5, expressed an understanding of sexual arousal and sexual acts. M2 explicitly associated the body with sexual excitement, commenting on the attraction between males and females (“It excites a lot when… when a male and a female do…”—M2 Pos 53) and identified anatomical features like the penis in this context. Similarly, F5 demonstrated an understanding of the sexual aspects of the body, associating sexuality with sensuality in her speech and readily confirming that her body could be involved in sexual activities and that such experiences could make her feel better (“I could feel better, feel good”—F5 Pos 686). Her statements indicate a simple but positive connection between sexual desire and emotional well-being. Furthermore, the experience of bodily pleasure was also localized by her in the body. When asked whether these emotions are felt somewhere in the body, F5 confirms: “Yes” (F5, 688). When asked where she initially answers generically: “Around here” (F5, Pos 693), and with the help of the interviewer identifies the area more precisely by referring to the intimate parts.

Nevertheless, not all participants made such direct connections between their bodies and intimacy. For example, when asked about feeling embarrassed sharing a bathroom with her father, F1 confidently responded, “No… Not at all” (F1, Pos 189), indicating that some individuals might not associate their bodies with sexual privacy in everyday situations. In summary, the psychosexual development of the participants involves a complex interaction of bodily awareness, gender recognition, and sexuality, with varying levels of understanding and emotional connection to these aspects of identity.

### 3.2. Theme 2: Affectivity, Relationships, and Self-Development

Relational and affective experiences play a central role in the construction of identity and self-esteem. In this regard, family relationships can serve either as a support or a conflict, influencing self-perception and independence. Romantic interactions, often characterized by idealizations, clash with the limitations imposed by familial and social contexts. Relationships with educational figures can also be both supportive and obstructive, impacting participants’ ability to develop effective emotional strategies and construct their adult identity. The main sub-themes that emerged include: (1) family relationships; (2) romantic relationships; (3) relationships with educators and secondary caregivers; (4) emotionality and self-development.

#### 3.2.1. Sub-Theme 1: Family Relationships

Family relationships play a fundamental role in shaping self-perception, emotional expression, and social identity. The participants’ narratives reveal a wide spectrum of experiences within their family units, ranging from affectionate bonds to conflictual dynamics. For some, the relationship with their mother is a source of emotional security, as M1 succinctly states: “Nice! (M1, Pos 181) when asked about his relationship with his mom. However, others experience cohabitation with their family as more ambivalent, with M2 describing the pleasantness of living with his mother and brother as “So-so” (M2, Pos 21). Tensions within the maternal relationship are also evident in certain cases, such as F7’s admission “Yes, I argue with mom”, and F4’s statement “I argued with her all the time.” (F4, Pos 371).

Sibling relationships can be equally complex, involving both affection and rivalry. F6, for example, describes her relationship with her brother as “so-so, not much,” explaining, “Because he makes fun of me, and I don’t like it” (F6, Pos 161). Conversely, some participants find a more stable and positive connection with their father, as F2 notes: “Yes, yes. I get along with my dad. Less so with mom, my sister, and my brother” (F2, Pos 237). These diverse experiences underscore how family relationships oscillate between moments of closeness and conflict, shaping the participants’ emotional development and social understanding.

The family also plays a crucial role in nurturing passions and shaping personal identity. M1 credits his grandfather and uncle with inspiring his love for music: “My passion is music… my grandfather got me into A. [Italian singer]” (M1, Pos 77), and “I’m really good! My uncle introduced me to jazz” (M1, Pos 381). Beyond fostering interests, family dynamics influence self-perception and adherence to social expectations. While F1 accepts parental authority unquestioningly—“If mom and dad tell me not to wear makeup, I say, ‘Okay, we’ll stay natural’.”(F1, Pos 318)—F2 expresses concerns about how parental decisions impact her individuality: “Mom likes me and twin sister to dress the same, but then it’s bad because it’s like we are the same person. If we wear different clothes, we are two different people” (F2, Pos 388).

Family relationships deeply affect emotional well-being, with some participants describing supportive connections while others face communication barriers and emotional distance. M1 reflects on his strained relationship with his father: “No! I don’t have a relationship with him anymore, I don’t talk to him” (M1, Pos 73). Symbolic objects sometimes serve as mediators for processing these relationships, as M1 explains: “I took this puppet... it made an impression on me because of its little face… it was scared, like me with my dad... but now it looks at me from my bed every night” (M1, Pos 133). For others, difficulties with paternal figures stem from personality clashes and habits, as F6 notes: “Because his character is too strong… I just can’t handle it. And he smokes, all the time—it smells awful” (F6, Pos 193).

Contrastingly, the mother–child relationship can be perceived as more harmonious, with F6 stating: “I get along better with my mom” (F6, Pos 185). Yet, maternal influence is not always perceived positively, particularly regarding autonomy. F2 expresses frustration when her mother chooses her clothes:

F2: Sometimes yes, sometimes no. We always argue about that.

I: How does it make you feel?

F2: Bad! (F2, Pos 374-376)

Sibling relationships, while often a source of support, can also be fraught with competition and tension. F1 describes the challenges of sharing a room with her sister: “It’s hard! If I ask to use the computer first, she says no. Same with the TV” (F1, Pos 49). When asked if they argue, F1 responds: “Yes, shouting,” and even admits to physical altercations: “Yes! And then mom and dad get involved. They say, ‘If you fight, I have to punish you both!’” (F1, Pos 57).

Despite these conflicts, some participants express pride in their siblings’ achievements, as M1 shares: “My sister graduated, and she dedicated her thesis to me!” adding, “Yes! I even wore my elephant-print tie for the occasion” (M1, Pos 393).

The participants’ accounts highlight the significant role of family relationships in their emotional and social development. Whether through moments of warmth or conflict, family interactions shape their sense of self, autonomy, and interpersonal skills. While some experience family as a source of security and encouragement, others navigate tensions that impact their confidence and decision-making. These relationships, in all their complexity, remain a crucial foundation in their journey toward self-awareness and independence.

#### 3.2.2. Sub-Theme 2: Romantic Relationships

The romantic relationships described in the interviews emerge through a complex interplay of fantasies, expectations, and lived experiences, shaped by both social influences and the pursuit of emotional autonomy. Love is often perceived through sensory experiences and tangible gestures that affirm the bond with a partner. Participants’ narratives reveal a spectrum of ways in which love is understood and experienced, ranging from dreams and idealizations to concrete relationship experiences.

A recurring theme is the representation of love through stereotypical imagery, emphasizing emotional intensity and the almost magical quality of romantic connections. F5 captures this sentiment: “Love is fantasy, because for me it is joy” (F5, Pos 99). Similarly, M3 describes love as a transformative experience: “Love... when love happens, it’s always between two people who want each other... the spark ignites, and then comes the kiss” (M3, Pos 130). When asked how one knows if the spark ignites, M3 explains: “You feel it in your stomach,” adding, “You feel butterflies” (M3, Pos 135).

Love is frequently described as a physical sensation, with bodily reactions serving as markers of affection and attraction. F1 elaborates: “Love is a very strong feeling you have when you’re with good-looking boys. When your heart beats really fast. When you have butterflies in your stomach” (F1, Pos 148). She further emphasizes the sensory nature of love: “And in the air… When you’re walking and you see a guy and say, ‘Wow, this guy is so handsome’… To win someone over, you have to catch him with your gaze” (F1, Pos 154). F5 echoes this idea, describing how her partner courts her: “He makes eyes at me, heart-shaped eyes” (F5, Pos 711).

The collective imaginary of love is also concretized through the notion of marriage, a theme that frequently emerges in the interviews. M4, for instance, shares his practical concerns about planning a wedding: “I need to save money for the ring. For the restaurant, for the church, for the house…” (M4, Pos 123).

The concept of love and being in love is frequently associated with physical expressions such as kissing and attraction. M5 succinctly defines love as “when you kiss someone” (M5, Pos 126), a sentiment echoed by M6, who explains how he chooses a partner: “With kisses” (M6, Pos 173). F4 also links the knowledge that she is in love to physical affection and contact, stating: “We kiss… we go to the seaside…” (F4, Pos 396).

Love is often understood in concrete terms, closely linked to visual and physical attraction. F2 explains how she recognizes being in love: “I like them… I only realize it when I see handsome boys because I’m obsessed with good-looking guys” (F2, Pos 563).

Family influence plays a crucial role in shaping relationship autonomy, as highlighted in multiple interviews. M1 confirms his mother’s approval of his romantic relationship: “Yesss, yes” (M1, Pos 365). Similarly, F5 notes: “Yes. She’s ok. I’m grown up” (F5, Pos 219). However, some accounts reveal disagreements and stricter parental control over romantic interactions. F7 describes her mother’s restrictions: “Mom says I shouldn’t stay on the phone with my boyfriend too long. ‘F7, you need to stay for a short time.’ I know, Mom, just a short time…” (F7, Pos 328)*;* M2, on the other hand, expresses uncertainty about his mother’s approval on his romantic relation: “I don’t know if she agrees” (M2, Pos 134).

The experience of romantic relationships is also shaped by personal and educational influences. M3, for example, frames love within a religious context: “Love is a religious thing because I believe in religion… I often go to church, and so does my girlfriend. We are on this spiritual journey together… Sometimes I’ve even gone on retreats with my church, looking for true love…” (M3, Pos 151).

These narratives illustrate a multifaceted understanding of romantic relationships, influenced by cultural expectations, physical attraction, family perspectives, and personal beliefs. Together, they paint a vivid picture of how love is imagined, felt, and navigated by the participants, reflecting both universal and stereotypical themes and individual nuances.

#### 3.2.3. Sub-Theme 3: Relationships with Educators and Secondary Caregivers

Relationships with educators and secondary caregivers play a crucial role in the emotional development and well-being of individuals diagnosed with intellectual and developmental disabilities (IDDs) or severe mental illness (SMI). The interviews reveal a mix of supportive interactions and challenges, particularly in communication styles, emotional responsiveness, and instances of misunderstanding.

Participants describe varying degrees of support and assertiveness in their interactions with educators but also highlight conflicts and frustrations. For example, F2 and F1 expresses dissatisfaction with her babysitter and the educators, stating:

I: How do you get along with the babysitter?

F2: With the babysitter? Terribly… she always bosses me around, just like the educators and my parents. (F2, Pos 228-229)

F1: My dad makes me do things too fast, like the babysitter, like the educators. I don’t get really along with them. (F1, Pos 45)

This suggests that the authoritative approach of secondary caregivers can feel oppressive, mirroring familial tensions and undermining the quality of the relationship. Similarly, F3 describes her frustration with educational tasks at the day center:

I: What don’t you like about the Center?”

F3: Doing homework.

I: Doing homework…

F3: Well, they gave me… they gave me a worksheet, and I do it… (F3, Pos 483–486)

The emotional responsiveness of educators is another contentious issue. While secondary caregivers attempt to address emotional needs, their approaches often seem misaligned with the complexity of the participants’ feelings. For instance, one participant recounts to the interviewer: “You see how they always treat me… they yell at me… like I’m a teddy bear. I get angry, I throw things around” (F3, Pos 647). Here, aggressive communication from the educators triggers an emotional outburst, highlighting how a lack of empathy can lead to frustration and impulsive reactions.

Relational difficulties and conflicts are evident in several accounts. Educators sometimes fail to effectively address the emotional needs of participants, leading to misunderstandings. M6, for example, expresses frustration when an educator insists on activities he no longer enjoys:

M6: How annoying, when she calls me.

I: What does she want from you?

M6: Well, you know, it’s been forever since I stopped doing theater

I: She wants you to do theater, but don’t you like it?

M6: For heaven’s sake, no! (M6, Pos 1301–1305)

Similarly, M1 explains that he cannot bring his puppet to the center to avoid upsetting an educator:

I: Can’t M1 bring the puppet?

M1: No. Because if the educator sees it, she’ll get mad. (M1, Pos 799–800)

When interactions are perceived as authoritarian or unwelcome, frustration becomes evident. F1, for instance, compares her secondary caregivers to military figures: “They make me work like a soldier,” (F1, Pos 76) referring to her babysitter and educators. She also describes the emotional toll of being scolded loudly at the center: “When they yell at me, I start crying” (F1, Pos 353).

All these episodes underscore how inadequate communication and authoritarian, infantilizing approaches can damage the quality of educational relationships, often leading to regressive emotional feelings and behaviors.

On the other hand, positive experiences with educators also emerge, particularly when activities align with participants’ interests and emotional needs. M1 fondly recalls outings with a former therapist:

M1: When I went out with my former therapist, he had me do some pretty cool things… like seeing the Van Gogh exhibit.”

I: Nice!

M1: “He took me bowling! (M1, Pos 599–601)

Such experiences demonstrate how meaningful activities and empathetic engagement can foster positive emotional connections and enhance well-being.

#### 3.2.4. Sub-Theme 4: Emotionality and Self-Development

Emotionality and the ability to recognize and manage emotions are central to self-development. The interviews reveal both challenges in processing emotional experiences and the strategies participants employ to navigate complex or negative emotions. Anger, in particular, emerges as a difficult emotion to manage. M1 describes his struggle with impulsive reactions in social situations: “It bothers me if someone touches me… I start getting angry like S. [Italian public person], I begin to do… strange things.” (M1, Pos 227). Similarly, F4 identifies anger as her most frequent emotion, explaining how it manifests physically:

I: Which of these emotions do you feel the most, anger, sadness, happiness…

F4: Anger.

I: And when you get angry, where do you feel it?

F4: Here!

I: In your head… and where else?

F4: In my legs.

I: The chest and legs…

F4: Yes, all over, all my torso and legs. (F4, Pos 377–383)

F1 also associates anger with physical sensations, stating:

F1: I feel anger when I get confused, when I have too many things to do and get tangled up, I get flustered.”

I: Where do you feel the anger? How do you know you’re angry?

F1: It happens because, when I get angry, I always do this [fidgeting movement with hands and frowning look]. (F1, Pos 817–819)

These accounts highlight how bodily sensations serve as key indicators of emotional experiences, particularly anger.

Fear is another challenging emotion, deeply affecting both the body and mind. F3 describes fear as something very impactful, often triggered by watching violent films and dramatic scenes that are difficult for her to process: “There were some scenes I didn’t like, like when they were saying next to them ‘I’m going to shoot you now’… then when I see the thing that the child cries, that… the scene that falls off the roof” (F3, Pos 561). F2 reflects on a past traumatic experience related to body care from experts:

F2: From the age of eight onwards, I have this phobia, I have this fear of taking care of my teeth, that as a child they put that white thing between my teeth to take care of my teeth.

I: And so…

F2: And I cried….

I: And now? And so even now you cry if you go?

F2: Yes, yes. Even now I happen to cry.

I: So… it’s not really a good experience this one.

F2: No, no, absolutely. (F2, Pos. 143-147).

However, not all participants can easily identify their emotions, as seen with M2:

I: What makes you angry?

M2: I don’t know. (M2, Pos. 24–25)

Conversely, positive emotions seem to be easier to recall and express, especially when it comes to love, as F2 notes:

I: And what did you feel on that occasion? Did you feel any emotion?

F2: Yes, definitely! Love… (F2, Pos. 534–535).

The process of constructing an adult identity intertwines self-perception, internalized values, and societal expectations. Participants express awareness of their growth and maturation, though their views of adulthood fluctuate between idealization, stereotypes, and lived reality. M1 describes himself as “a bit of a comic guy” (M1, Pos 199), while M2 identifies as “a kind man” (M2, Pos 77). M3 reflects on balancing maturity with childlike qualities: “I’m an adult, but I also try to be like the kids… but I still try to be myself… I’m grown” (M3, Pos 36) and questioned about what it means, he answered: “Being a man means being more… open.” (M3, Pos 42).

For others, adulthood is tied to behaviors and responsibilities. M4 defines himself as “a serious man,” explaining: “one that works.” (M4, Pos 31–35). F7 similarly associates adulthood with proper behavior: “An adult means someone who behaves properly […] You shouldn’t swear or do those things. And I swear…” (F7, Pos 60–64).

Achieving milestones tied to societal ideals also marks the transition to adulthood. F5 expresses her desire to marry:

F5: First thing, I want to get married because now I’m grown.

I: When did it happens?

F5: I’m grown because… my age surpasses my height.

I: And what grown-up things do you want to do?

F5: I want to shop, cook… feed children, have a boyfriend. (F5, Pos 245–249).

Physical changes further signify growth, as F1 explains:

I: Did you understand why your mom made you wear a bra?

F1: Yes, because I’m slowly growing, my chest is developing.

I: So, your mom explained what was happening?

F1: Yes.

I: And how did you feel about it?”

F1: I felt great because I felt just like my mom. (F1, Pos 761–767).

Personal growth is also reflected in affective relationships and emotional awareness. F2 describes how her understanding of relationships evolved with age: “When I was younger, I only considered my friends just friends because we were too young to understand emotions. But as we get older, we quickly realize what love is.” (F2, Pos 561).

Social interactions and the development of meaningful relationships are central to self-development. However, individuals with intellectual and developmental disabilities (IDDs) or severe mental illness (SMI) often face significant challenges. F1 recounts bullying during high school:

“High school was really hard because I had no relationships with classmates since I was little until I grew up. They kept bullying me, treating me badly, throwing things at me, like wet rags, for example, while I was in the bathroom doing my business, even when I changed my pad, my friends… they took the bathroom rags, filled them with water, and threw them at me, all the way up to here.” (F1, Pos 39–41).

M1 similarly shares past teasing:

M1: They called me a banana helmet when I was little.

I: Who called you a banana helmet?

M1: …oh, they used to tease me… ’oh, M1 has a banana helmet on his head.’ I was also a bit of a target, sometimes they’d make me bang my head against windows, they’d make me angry… (M1, Pos 279–283).

Despite these challenges, participants continue to seek and build meaningful connections. M1 finds joy in making others laugh: “I make everyone laugh when I tell a joke!” (M1, Pos 201). M5 highlights his social network: “I have a lot of friends, I have A. M.” (M5, Pos 100). F7 describes her positive connection with a peer: “I sit by the computer, we socialize, we talk, and I feel good with F4, I have to say the truth.” (F7, Pos 505). However, some participants exhibit confusion regarding roles and relationships, as seen in M2’s response:

I: Do you think you are in a relationship or it is just friendship?

M2: I don’t know. (M2, Pos 125–126).

Another participant, F4, claims to be in a relationship with a staff member: “G. is my boyfriend” (F4, Pos 143).

These narratives highlight the intricate interplay between emotional experiences, self-perception, and personal growth. While anger and fear emerge as particularly challenging emotions to navigate, often manifesting through physical sensations, positive emotions like love are more readily recognized and expressed. The journey toward adulthood is marked by a blend of self-awareness, societal expectations, and milestones, with participants reflecting on their evolving identities and responsibilities. Despite the emotional and social challenges they face—ranging from traumatic experiences to bullying— many participants demonstrate resilience and a desire to build meaningful connections. These accounts underscore the importance of supportive environments that foster emotional well-being, autonomy, and self-development, enabling individuals to navigate their emotions and relationships with greater confidence and clarity.

### 3.3. Theme 3: Gender Identity and Sexual Orientation

Gender identity and sexual orientation are central dimensions of personal identity, emerging through experiences, perceptions, and social influences. Some individuals have a clear awareness of their identity and attractions, while others exhibit uncertainties or a more fluid understanding. Social roles and gender stereotypes impact the possibilities for individual expression, with expectations that may limit personal choices. Knowledge of and engagement with LGBTQ+ issues vary, with some participants showing openness and others remaining influenced by familial and social norms. The main sub-themes that emerged include: (1) sexual orientation and attraction; (2) perception of gender identity; (3) social roles and gender stereotypes; (4) knowledge of and relationship with LGBTQ+ issues.

#### 3.3.1. Sub-Theme 1: Sexual Orientation and Attraction

The recognition and understanding of one’s sexual orientation emerged at various points in the analysis, illustrating the diverse ways in which self-awareness develops. For some participants, this recognition appears to be immediate. F1, for instance, states: “I liked him right away” and when asked directly, “Do you like boys or girls?”, she responds without hesitation: “Boys.” (F1, Pos 660–661). Likewise, M1 firmly asserts: “I’m not a gay guy.” (M1, Pos 1683).

In some cases, expressions of sexual orientation appear to be framed within rigid categories, possibly influenced by cultural expectations:

I: (…) It depends, a girl can also choose to date girls, she doesn’t have to date compulsory boys. But what does F7 prefer?

F7: I have to choose a boy, not a girl. (F7, Pos 417–418).

This statement may suggest an internalized norm rather than a freely articulated preference. Similarly, in another participant sexual orientation is expressed with rigid certainty, as in F2’s statement: “Boys. I’m not a lesbian, I’m not homosexual. I’m not bisexual, I’m heterosexual” (F2, Pos 538).

Conversely, F4 expresses a more fluid perspective, stating simply: “Both.” (F4, Pos 234), indicating an openness to attraction regardless of gender.

Expressions of romantic and sexual attraction also emerged with clarity, often tied to a variety of experiences. When asked, “What do you like about a girl?”, M2 replies: “The body” (M2, Pos 68). Driven further—”What exactly?”—he specifies: “The legs” (M2, Pos 68). Similarly, M1, when asked about his preferences, replies: “I like looking at a woman’s body, you know. Or another thing—I like seeing tattoos” (M1, Pos 1207). On the other hand, F5 offers a more general perspective, stating: “Everything… the lean body” (F5, Pos 117), when asked what she likes about her partner. F7, too, expresses attraction in a straightforward manner, stating: “The body.” (F7, Pos 233), as the defining aspect she finds appealing in her boyfriend.

These accounts highlight the diverse ways in which sexual orientation and attraction are perceived, articulated, and experienced among participants.

#### 3.3.2. Sub-Theme 2: Perception of Gender Identity

The awareness and self-definition of gender identity emerge clearly in participants’ responses, with some demonstrating a strong and confident sense of self. F2 firmly states, “I am a girl,” (F2, Pos 597), while M5 confidently identifies as “A boy.” (M5, Pos 154). These straightforward declarations reflect a secure understanding of their gender identities.

However, other responses suggest a more complex or evolving understanding of gender. F5 initially hesitates, stating, “Male, no, a female… I am a woman,” (F5, Pos 741), indicating a moment of reflection before affirming her identity. Similarly, M1’s response to a question about gender presentation reveals a playful yet uncertain perspective:

I: I have a doubt… if a male dressed as a woman, did he become a woman?

M1: Yes, he became a woman. (M1, Pos 853)

Participants also express a strong awareness of gender differences, particularly in terms of physical attributes and social roles. F2 provides a detailed description of these differences: “Well, men grow sideburns, a beard, mustache, and a goatee. For us, it’s the breasts, then we get our period. And then we women have to do things like waxing, hair!” (F2, Pos 603). F7 elaborates her perception about gender differences on hygiene practices:

F7: Boys grow hair… they use deodorant, wash themselves… those things, like washing. Girls wash, take care of themselves… eyebrows, mustache, those things.

I: And do boys do their eyebrows and mustache?

F7: Nooo, no no, they just use a razor. (F7, 199–207).

F1 describes how gendered behaviors are reinforced through media and clothing:

F1: For example, girls buy girls’ clothes, wear high heels, watch girls’ cartoons, and watch movies for girls, like ’The World of Patty’ and ’The Incorrigibles.’ And how do I know if you are a boy? The same way, you buy boys’ clothes, and then what do you do? You smoke, you watch boys’ cartoons… (F1, Pos 931)

These responses highlight how gender identity and roles are often shaped within a cultural framework. While some participants articulate their gender identity with clarity, others navigate predefined categories influenced by social expectations regarding physical appearance and behaviors. Together, these narratives illustrate the diverse ways in which gender is understood, experienced, and expressed by the participants.

#### 3.3.3. Sub-Theme 3: Social Roles and Gender Stereotypes

The analysis of social roles and gender stereotypes reveals a clear distinction between behaviors and expectations related to dating, marriage, and parenthood, each linked to traditional gender norms. Dating is described as a phase where gender roles begin to take shape, with distinct behaviors associated with males and females. F1 highlights the expectation that men initiate romantic relationships: “I think the boy can do it first, because he makes the first move” (F1, Pos 879). Similarly, F5 describes courtship as a male-driven activity: “I’m the woman. He courts me.” (F5, Pos 281).

Marriage is viewed as a more serious commitment, consolidating traditional gender roles. F1 explains the progression from dating to marriage: “Being a boyfriend is when you are young […] then, if you like him so much, you marry him and become husband and wife.” (F1, Pos 1345). She elaborates on the social expectations surrounding marriage: “When you are married, you start visiting each other’s houses, meeting each other’s families. If they ask questions like, ’How long have you been together?’ or ’Why are you together?’, then… then you don’t become husband and wife anymore with divorce” (F1, Pos 1345). F5 associates marriage with happiness and tradition: “Marriage is happiness... I want to live alone with my husband, have a house, do many things, have wedding favors, a restaurant” (F5, Pos 664).

Parenthood, often seen as the next step after marriage, is described as a joint commitment with a clear division of roles. M5 links marriage to parenthood:

I: What does it mean to become a wife? Do you know what it means to have a wife?

M5: To have many children. (M5, Pos 210).

F5 views motherhood as both challenging and sacred: “It is heavy, but it is necessary in life… a gift to me, for affection, a gift given by Jesus” (F5, Pos 755).

Gender roles are shaped by familial, social, and cultural influences. F2 explains how her family taught her that girls must be beautiful to attract boys:

F2: I think it is necessary to attract a boy, like when they put on makeup, dress up, wax, do their nails and pedicure.

I: Who told you that?

F2: Mom and Dad. (F2, Pos 121).

Conformity to gender stereotypes extends to clothing and social norms. M1 expresses disapproval of women wearing suits and ties: “I hate… Ah, one thing I hate is women in suits and ties. I hate them… Only men can dress that way. Only men, because I am a man, but not women. Pink ties? What is that? It looks like a fashion trend from two years ago, but are we going back to that?” (M1, Pos 1602). F1 reveals a racial bias in her preferences for relationships: “I like black people, but I like making love with white-skinned people, marrying white-skinned people, and having white-skinned children. Because if I get yellow-skinned ones, black-skinned ones, I say nooo I don’t want them, I don’t like it, no!” (F1, Pos 1006). M3 reinforces traditional family structures: “The man must be the man, the center of the family.” (M3, Pos 52).

Gender roles are also reflected in domestic work and employment:

F6: I’m a girl, a little girl.

I: And what does it mean to be a girl?

F6: Do house chores: ironing clothes, helping my mom with the dishes, loading the dishwasher, cleaning the whole house

I: And boy? What do boys do?

F6: They work. (F6, Pos 75–80).

These narratives illustrate how traditional gender roles and stereotypes are deeply embedded in social expectations, influencing behaviors and perceptions across dating, marriage, parenthood, and domestic and work life.

#### 3.3.4. Sub-Theme 4: Knowledge of and Relationship with LGBTQ+ Issues

Participants demonstrate a partial understanding of LGBTQ+ issues, with some grasp of key concepts but also noticeable terminological confusion. F1, for example, correctly describes transgender individuals as those who “transition from female to male, or from male to female,” (F1, Pos 935) showing basic awareness of gender transition. However, she misinterprets the LGBTQ acronym:

F1: LGBT means that all people who are trans must love, they have to love.

I: So, LGBT only counts for trans people?

F1: Yes, just for trans people. LGBT is a community where there are all those trans people you see on the street who say you have to learn to love trans people. I heard it on ‘Catfish’. (F1, Pos 1002–1004).

Some participants exhibit a more nuanced but still uncertain understanding of same-sex relationships. M2 suggests that same-sex intimacy may be “for fun,” (M2, Pos 111) adding, “To get excited too” (M2, Pos 111). F2 acknowledges that same-sex couples can have intimate relationships despite not being able to procreate:

F2: They can have sex, but they can’t have children in the belly.

I: And why, according to you, do they do it?

F2: I think they do it for love. (F2, Pos 552).

Attitudes toward LGBTQ+ individuals range from acceptance to strong prejudice. F4 expresses openness to same-sex relationships:

F4: A woman and a man.

I: Ok, a woman and a man, but what about two women?

F4: Two women…

I: Who are in a romantic relation. Is that possible?

F4: Yes, yes.

I: And two men in a romantic relation?

F4: Too. (F4, Pos 242–248).

Conversely, others reject the idea, adhering to traditional views on sexuality and family structures. F2 states:

F2: Men and women.

I: Only?

F2: Only. Not man and man, woman and woman. It disgusts me. No, and I don’t like gay marriages either. (F2, Pos 540).

Similarly, F5 dismisses the possibility of same-sex intimacy: “No, no, not that. I don’t like it” (F5, Pos 829).

M1 exhibits conflicting views, oscillating between rejection and acceptance. Initially, he expresses strong aversion:

M1: I don’t like seeing a guy kiss another guy, it disgusts me.

I: What bothers you?

M1: A girl kissing another girl made me want to hit myself, to punch myself! It really upset me! (M1, Pos 400–401).

However, later, his stance appears more accepting:

I: If a girl kisses a guy on the street, does it bother you?

M1: No, no.

I: And if a guy kisses another guy?

M1: No, no, It’s ok. I even have a family member who is like that. (M1, Pos 410–411).

These responses underscore the influence of personal experiences, social conditioning, and cultural norms on participants’ perceptions of LGBTQ+ issues. While some exhibit openness and acceptance, others reveal deep-seated biases, highlighting the need for inclusive education and dialog to foster greater understanding and reduce prejudice. Together, these narratives illuminate the diverse and often contradictory ways in which individuals navigate and relate to LGBTQ+ identities and relationships.

### 3.4. Theme 4: Sexuality

The exploration of sexuality among the participants is shaped by a range of factors, including fragmented information, misunderstandings, and gaps in sexual education. Their understanding of sexual practices and intimate behaviors is often inconsistent, with some participants holding misconceptions or incomplete knowledge, highlighting the lack of structured sexual education tailored to their cognitive and emotional needs. Sexuality is frequently understood in narrow terms, primarily linked to reproduction, with the belief that sexual activity occurs only within marriage and solely for the purpose of having children.

The theme of intimacy is marked by a mixture of curiosity, discomfort, and anxiety. For many participants, the notion of sexual intimacy triggers conflicting emotions, ranging from curiosity to fear, and sometimes even disgust. Some express clear personal boundaries regarding certain intimate acts, which point to the emotional and psychological complexities that accompany the exploration of their sexuality.

Sexual education is further influenced by external factors such as access to the media, pornography, digital platforms, and cultural or familial beliefs. The interviews reveal a fragmented and often distorted understanding of sexuality, shaped by unreliable sources and cultural norms that limit or skew their perception of their own bodies and relationships. Education from family or caregivers is often minimal or non-existent, with information conveyed primarily through prohibitions rather than open discussions. The main sub-themes identified are: (1) sexual practices, fantasies, and behaviors; (2) intimacy, desire, and emotional tensions; (3) sexual education and cultural influences.

#### 3.4.1. Sub-Theme 1: Sexual Practices, Fantasies, and Behaviors

Participants’ understanding of sexuality appears fragmented, shaped by limited education, misconceptions, and knowledge gaps. Some participants struggle to articulate what sexual intimacy entails, resorting to vocal sounds or mimicking gestures of pelvic movements, while others express vague ideas on the subject:

I: What can boys and girls do together if they are in an intimate relation?

F7: Love… they make love…

I: And what does making love mean?

F7: I don’t know, it’s talking, maybe… (F7, Pos 334–336)

I: Have you ever heard about making love?

F6: Yes, in a movie.

I: Which movie?

F6: The movie is ‘Sorry, If I Call You Love’

I: Ah… and what does making love mean to you?

F6: That a girl gets engaged.

I: And that’s making love?

F6: Yes. (F6, Pos 168–175).

Others display a complete lack of exposure to sexual knowledge:

I: Do you know what sex is?

F4: No.

I: Have you ever heard about it?

F4: No, never. (F4, Pos 463–467)

Sexuality is often linked exclusively to reproduction, with some believing that sex should only occur within marriage and for the purpose of having children:

I: What does making love mean?

F2: Once you are married, you make love—when you want to have a child. (F2, Pos 506)

However, some demonstrate awareness of contraceptive methods, albeit with confusion:

F1: Some people take ‘condoms’ so, they don’t have children while having intercourse.

I: Do you know what a condom is? Have you ever seen one?

F1: No, but I think it’s those pills, like medicine, that you take by mouth before having sex. (F1, Pos 871–873)

Even when knowledge is present, it is often characterized by strong information:

I: How does it work to ‘make love’?

M6: Someone gets on top, a man gets the pecker.

I: OK, and what happens?

M6: Where does he put it, you mean? Behind, behind the ass and that is making love. (M6, Pos 782–784)

Sexual experiences among participants range from curiosity and exploration to a more direct engagement in intimacy. Some accounts depict physical closeness between partners:

I: Why did you take off your clothes? What did you want to do?

F3: Hands on my breasts… and he kiss me in the mouth and also [she indicates the breast]… and bruise-like marks appear… do not come off. (F3, Pos 316–318).

Some participants recall the beginning of sexually explicit encounters during a holiday:

I: You undressed, what did you take off?

M3: First the trousers, trousers and the shirt.

I: So, you stayed in your underwear?

M3: Exactly… then the educator went up the stairs opened the door and there…. he found me on top of her…. and…. right in that room we had started to have sex and… that room is cursed because… the educator separated us.

I: But how was it?

M3: For me it was nice, but for the educator not so much. I felt happy, but as soon as he came in… (M3, Pos. 232–247)

These two accounts underline the tension between sexual curiosity and exploration, and the external forces (such as societal norms, institutional authority, and disability-related prejudice) that often shape or limit the participants’ experiences. They also shed light on the emotional and psychological dimensions of these encounters, where pleasure, shame, and institutional control are deeply intertwined

For some, sexual intimacy remains an unexplored concept:

I: Have you ever made love to someone?

M2: No. (M2, Pos 76)

Pleasure and bodily sensations also emerge as themes in participants’ reflections:

I: Do you like being touched, or does it bother you?

F5: No, it doesn’t bother me.

I: Even when you’re naked?

F5: Yes, I like it. (F5, Pos 342–345).

The theme of sexual fantasies and unexpressed desires emerges alongside participants’ accounts, oscillating between curiosity, confusion, and idealized romantic imagery, especially in female participants. Many express a desire to learn about intimacy but struggle to conceptualize it beyond simplified notions, often influenced by fairy-tale-like narratives and idealized relationships. F5, for example, describes one of her fantasies: “He makes love to me… so he touches me… he touches my breasts… he puts me under… he kisses me. […] I talked to him about this fantasy, but first marriage” (F5, Pos 789 and 795).

Participants also express a clear wish to learn more about affective and sexual relationships. F2 states: “I would like to learn how to get married, and… also always how to kiss with the tongue and learn how to have sex, learn how to have children.” (F2, Pos 819). Similarly, F1 shares her curiosity which she would like to explore further: “I’d like to know things about sexuality… how babies are made, I like being engaged, being married, how to become a husband and wife, what relationship and what bond exists between fiancés and between husbands and wives.” (F1, Pos 1343).

Overall, participants’ perceptions of sexuality reflect a complex mix of curiosity, misinformation, and varying degrees of personal experience. Many participants express idealized fantasies and misunderstandings about intimacy, often shaped by fragmented or distorted sources.

#### 3.4.2. Sub-Theme 2: Intimacy, Desire, and Emotional Tensions

The management of sexual desire and intimate situations is often complex and laden with conflicting emotions. For many participants, the lack of clear and appropriate sexual education leads to feelings of confusion, frustration, or even rejection of sexuality. Some struggle to articulate their thoughts, as seen in M1’s response to advice from a doctor: “I don’t like when… my doctor told me to look at naked girls… I don’t know…” (M1, Pos 567).

Intimate experiences, such as kissing, are often described positively but simultaneously generate uncertainty and the need for boundaries. F7 reflects on a past experience:

I: What do you think, when, for example, you kissed your boyfriend, was it a nice thing or…?

F7: It was a nice thing. I liked it, but I don’t want to do it anymore, sometimes… but not always, no, I don’t want to do it all the time! (F7, Pos 339–340).

For some, sexual desire intertwines with emotions of anger and frustration, leading to aggressive or rejecting reactions. M1 expresses strong discomfort with exposed sexuality:

M1: It’s something I hate, it makes me want to punch people.

I: Punch them?

M1: Punch, I mean, punch the women. I don’t like seeing someone undress half-naked and show their breasts. (M1, Pos 1572–1574).

This reaction may stem from a sense of inadequacy or difficulty managing excitement and desire.

The decision to avoid relationships can also arise from a need for protection and tranquility. F6 explains her preference for solitude:

I: Does F6 ever had a boyfriend?

F6: No, never.

I: Why not?

F6: Because… I like being alone, it’s quieter… it’s better to be calm, that’s all. (F6, Pos 90–93).

Fear of the unknown, especially regarding physical intimacy, is a recurring theme. F2 shares her anxiety about kissing: “Then when I have to date and marry, I’m afraid to kiss him with my tongue, because I don’t know how to do it, I don’t know how to kiss with my tongue…” (F2, Pos 699). Later on, F2 expresses deeper concerns about genital sexuality: “I’m afraid to do it, I don’t know if it’s okay or not. I don’t know if I can have children or not. I’m afraid I’ll become sterile. But I don’t want to be sterile, I want to be fertile.” (F2, Pos 695).

The exploration of sexuality is often accompanied by feelings of anxiety, discomfort, and uncertainty. Some participants clearly express rejection or personal limits regarding certain intimate acts. M6, for example, sets a boundary:

I: Does M6 want to do something like that?

M6: Noooo, no, I just want to kiss my girlfriend. (M6, Pos 865–866).

Physical contact can also evoke discomfort or disgust. F3 describes her aversion to certain gestures:

I: How do you feel when your boyfriend puts his hands in your breasts?

F3: I feel bad. It is bad. (F3, Pos 750–751).

A sense of shame and reserve also surfaces in relation to affectionate gestures. F6 explains her hesitation:

I: Do you hug and kiss sometimes the boy you like?

F6: No, just here. [She indicates the cheeks]

I: Why just on the cheek?”

F6: Because it makes me feel ashamed. (F6, Pos 136–139).

These narratives highlight the complex interplay between intimacy, desire, and emotional responses. While curiosity and affection play significant roles in shaping these experiences, they are often overshadowed by anxiety, fear, and societal constraints. Together, they illustrate the multifaceted ways in which participants navigate intimacy, balancing their desires with emotional and social challenges.

#### 3.4.3. Sub-Theme 3: Sexual Education and Cultural Influences

Sexual education among participants appears fragmented, shaped by a mix of media exposure, pornography, family influences, and cultural beliefs. The lack of structured sexual education, often replaced by prohibitions rather than explanations, is evident across the interviews. For instance, one participant recalls: “As soon as [the educator] entered… he separated us because I’m a Down boy, and she’s a Down girl… two Down individuals can’t have sex…” (M3, Pos 247). When asked if this was a personal belief or something told to him, he adds, “I think it was right… anyway, we don’t have the age” (M3, Pos 257).

Another participant expressed a lack of education on sexuality in her family:

I: Did they explain what sex is? How to do it?

F1: No. (F1, Pos 860)

And further clarified that the only rule passed on by her family was that “sex is only for people engaged” (F1, Pos 862).

Similarly, some participants report unclear or incomplete information from family members:

F2: I heard about these three types of sexuality from my dad. Oral sex, physical and psychological.

F2: Did he explain what they are?

F2: No. (F2, Pos 669–671).

Another recalls a parental restriction on sexual practices:

I: …and your mom, does she know you’re in a relationship?

F3: No… and she doesn’t allow me… the kisses… because he makes signs on my chest… she told [the educator] ‘why does F3 do these things?’ (F3, Pos 308–309).

Some participants, however, have received more direct, albeit intrusive, guidance from professionals: “There’s one thing I don’t like, when my therapist says, ‘touch yourself!’ I really hate it!” (M1, Pos 505).

This statement suggests that professional interventions in the domain of sexuality are not always welcomed or perceived as appropriate. In this case, M1 expresses clear discomfort, suggesting that the guidance received was intrusive rather than supportive. This could indicate a lack of sensitivity to individual boundaries or personal readiness for discussions about sexuality. Whether the professional’s intent was to promote bodily awareness or encourage exploration, M1’s response underscores the importance of consent, emotional readiness, and the need for a tailored approach in sexual education for individuals with intellectual and developmental disabilities.

In the absence of structured sexual education from family or professionals, some participants turn to pornography as their primary means of learning about sex. One participant explicitly states that neither his mother nor his siblings ever explained what sex is, leading him to seek out information independently:

I: And where did you learn?

M3: I learned by myself.

I: What did you use to learn?

M3: I watched… because I watch videos… porn.

I: When do you watch them?

M3: On my phone. (M3, Pos 282–289).

This highlights how pornography serves as an informal source of sexual knowledge, often shaping participants’ perceptions, despite its limitations in providing a comprehensive understanding of intimacy and relationships. While some participants actively engage with such content, others describe exposure to pornography through friends or family members:

M6: I used to see it too on my cousin’s mobile phone. It is better not to see.

I: No?

M6: No, it’s disgusting. (M6, Pos 803–805).

Similarly, another participant recounts a brief exposure to pornography and her negative reaction:

I: So, your friend showed you these videos…

F1: Yes. And I told her, ‘Let me see.’ But after just one video, I had seen enough of that. What is this pornography thing?

I: You didn’t like it?

F1: No. No? What does this pornography thing even mean?

I: Didn’t you like what you saw?

F1: No, no, because even in movies they have sex scenes, or even in Japanese cartoons I’ve seen sex scenes.

I: How was that?

F1: The cartoon ones were nice. For example, I liked ‘Kiss Me Licia’ because, during the opening theme, you see Mirko and Licia getting married and making love. (F1, Pos 475–483).

Exposure to pornography does not necessarily lead to its acceptance or use as a positive or desirable model for sexual education; it can also provoke rejection or disgust. M6’s reaction underscores that exposure does not equate to endorsement, highlighting the need for structured sexual education that goes beyond mere access to explicit content. Instead, F1 prefers romanticized depictions of intimacy, such as those in animated series, which she describes in positive terms. This further illustrates that participants do not passively absorb all media representations but instead develop their own preferences and boundaries regarding sexual content.

Some have been exposed to such content from a very young age:

I: Do you know what pornography is?

F2: Yes. It’s those movies with all the nude sex scenes.

I: Have you seen them?

F2: Yes, when I was little… since I was 7. (F2, Pos 684–687).

Other participants, however, show little to no interest in pornography:

I: Do you ever watch videos on your phone of people having sex?

M2: No… I’m not interested. I think it could be a bit violent. (M2, Pos 117–118).

Beyond pornography, digital platforms and online searches play a significant role in sexual learning: “I search for romantic sex videos on my phone. Typing sex, romantic, fiancés…” (F5, Pos 776). Similarly, media, including films and advertisements, shape perceptions about sexuality and are perceived as sources of information and education. One participant shared: “I learned from movies, watching how it’s done—first you undress, then you touch and kiss the person’s body, then I saw in advertisements that before having sex, you need to put on deodorant on intimate parts too” (F1, Pos 865).

Moreover, some participants gain information from educational materials like books: “I learned from this book [name of the book]” (M2, Pos 122). Another participant explains a scientific description learned in school: “In science class… I learned how sex is done. You put the penis in the pubic area, and then sperm enters the egg, and that’s how a baby is made” (F2, Pos 514).

The combination of fragmented sexual education, media exposure, and cultural influences creates a distorted and sometimes incomplete understanding of sexuality, leaving participants to navigate complex emotional and relational landscapes with limited guidance.

## 4. Discussion

This exploratory study underscores the intricate challenges young adults with severe mental illness (SMI), intellectual disabilities (IDs), and autism spectrum disorder (ASD) face in navigating body perception, affectivity, intimacy, gender identity, and sexuality. The findings reveal systemic barriers rooted in societal norms, familial practices, and institutional gaps, while highlighting participants’ resilience and agency in negotiating these complexities. These barriers reflect broader patterns of structural inequity, where ableism, heteronormativity, and diagnostic overshadowing intersect to marginalize individuals with disabilities [35,60]. The results align with prior research on the systemic exclusion of disabled populations from sexual health discourse [4,28], yet they extend this literature by elucidating how these exclusions manifest across micro-, meso-, and macro-level systems—a framework informed by Bronfenbrenner’s ecological theory [13,14,15] and Prilleltensky’s [9,10,11,12] critical psychology lens.

A central finding is the tension between bodily autonomy and familial control. Prolonged dependence on caregivers for intimate hygiene, such as bathing or menstrual management, underscores the pervasive infantilization of individuals diagnosed with SMI, ID, and ASD, often rationalized through narratives of vulnerability [7,19]. Prilleltensky’s theory of well-being and justice [9] enables us to frame these practices as violations of psychological needs for competence and self-determination, a perspective that aligns with other research [20,36], demonstrating how families of individuals with disabilities may prioritize risk aversion over autonomy, inadvertently reinforcing cycles of dependency. However, instances of self-advocacy—such as asserting independence in personal care—highlight the potential for structured interventions that balance familial support with skill-building programs tailored to individual readiness. These findings echo broader calls [24,30] for family-centered psychoeducation that reconciles safety concerns with rights-based approaches.

Body image struggles, shaped by familial critiques and gendered expectations, further illustrate, in line with the literature [2,22], how external pressures internalize stigma, particularly for those navigating intersecting identities (e.g., disability and femininity). Here, Prilleltensky’s macro-level analysis [11,12] contextualizes how societal beauty standards and ableist norms exacerbate these struggles, while Bronfenbrenner’s [14,15] exosystem (e.g., media representations) explains their infiltration into familial interactions.

These dynamics intersect with relational and gender norms. Familial relationships, while often a source of emotional stability, simultaneously restricted participants’ exploration of identity, particularly for those inquiring about gender or sexual orientation. Adherence to heteronormative scripts, reinforced through parental teachings and cultural narratives, marginalized non-conforming identities and limited access to LGBTQ+-inclusive resources. This mirrors prior findings [1,22] on the “double stigma” faced by LGBTQ+ individuals with disabilities, but it also extends them by highlighting Bronfenbrenner’s [13,14,15] chronosystem—how delayed access to inclusive education perpetuates intergenerational cycles of misinformation. Romantic idealizations, influenced by media tropes and religious frameworks, often lacked grounding in practical guidance on consent or boundary-setting, exacerbating vulnerabilities to exploitation or miscommunication [24,50]. Prilleltensky’s critique of organizational injustice [12]—which highlights how institutions often perpetuate systemic inequities rather than addressing social needs—elucidates how meso-level systems (e.g., schools, rehab centers) fail to bridge this gap, leaving families as the sole—and often ill-equipped—sources of sexual socialization [28,33].

Similarly, interactions with educators oscillated between supportive engagement and authoritarian oversight, underscoring the need for trauma-informed training to dismantle power imbalances and foster agency [65,66,67,68]. This aligns with the literature on stigmatization and microaggressions in mental healthcare [65], while Bronfenbrenner’s meso-system framework [13,14,15] helps to contextualize how tensions between institutional structures and interpersonal relationships shape service users’ experiences of exclusion, exacerbating power imbalances.

Participants’ understanding of sexuality was fragmented, shaped by unreliable sources such as pornography, peers, or sensationalized media rather than structured education. Misconceptions about contraception and sexual health—such as conflating condoms with oral medication—expose systemic failures in providing accessible, disability-adapted curricula [6,28]. Prior research [4,14] identifies these gaps as endemic in disabled populations. Our findings further connect them to Prilleltensky’s concept of distributive injustice [9,11,12], emphasizing how systemic inequities and interpersonal dynamics restrict access to essential resources and opportunities contributing to psychological distress and the possible failure of social policies. Additionally, Bronfenbrenner’s macro-system framework [13,14] helps to contextualize how broader policy decisions and societal attitudes perpetuate the marginalization of disabled perspectives.

While curiosity about intimacy was common, internalized shame and fear of stigmatization often deterred individuals from seeking information or forming relationships, perpetuating cycles of isolation [27,29]. These results corroborate studies [4,7,8,21] linking poor sexual education to victimization risks.

The analysis reveals relevant variations across gender, diagnosis, and independence levels that both confirm and extend the existing literature. Women experienced greater familial control over bodily autonomy, particularly regarding hygiene and appearance [19,22], reinforcing previous findings about gendered socialization in disabled populations [2,23]. Conversely, men faced rigid gender expectations despite greater self-care independence [18], with their greater focus on physical attraction and pornography exposure aligning with patterns observed in broader disability studies [18,23].

Diagnostic differences further nuanced these patterns. Individuals with IDs showed prolonged caregiver dependence and romanticized relationship concepts, consistent with documented gaps in sexual education for this population [19,26]. Those with SMI encountered pronounced social stigma limiting relational opportunities [17,35], while ASD participants’ boundary challenges and attraction patterns validated prior work on autism and sexuality [7,23].

The autonomy continuum proved particularly significant, with independent participants demonstrating greater sexual agency—a finding that extends Andreassen et al.’s [28] work on disability and self-determination. However, although these trends suggest meaningful differences, the small sub-group sizes prevent definitive conclusions about whether these distinctions reflect generalizable patterns or individual variability [69]. Future research with larger, balanced samples remains crucial to systematically explore these dynamics and develop targeted interventions.

### 4.1. Implications for Future Supportive Interventions

These findings demand a paradigm shift in supporting young adults with SMI, IDs, and ASD—one that transcends current compartmentalized approaches. Building on Prilleltensky’s [9,10,11,12] emphasis on justice-oriented praxis, interventions must simultaneously address micro-level autonomy needs and macro-level structural barriers.

Educators, caregivers, and policymakers must prioritize autonomy-building by replacing authoritarian or infantilizing practices with collaborative frameworks that honor individual agency. This includes co-designing skill-building programs for self-care and decision-making, tailored to the unique developmental trajectories of individuals with intellectual disabilities, autism, and SMI, while addressing familial anxieties about risk and vulnerability. Such programs could draw on gradual transitions to independence, peer mentorship, and family workshops that reframe dependency narratives into empowerment opportunities.

Equally critical is the urgent need for inclusive sexual health education that bridges systemic gaps in knowledge and access. Current reliance on fragmented sources like pornography or peer networks perpetuates misconceptions and vulnerabilities. Comprehensive, disability-adapted curricula must address cognitive needs, use accessible language, and integrate LGBTQ+ perspectives to dismantle internalized stigma and heteronormative assumptions. These efforts should be coupled with trauma-aware approaches to discussions about consent, boundaries, and intimacy, ensuring individuals are equipped to navigate relationships safely and confidently.

At a systemic level, institutional reforms must dismantle structural inequities by fostering interdisciplinary collaboration between mental health services, disability advocates, psychologists, researchers, and educators who are experts on the subject. Funding for community-based programs should mandate trauma-informed certification for professionals, emphasizing anti-ableist practices and harm reduction. Policymakers must also amplify lived experiences by centering individuals with SMI, IDs, and ASD in advocacy and program design, ensuring their voices shape policies that directly impact their lives.

**Figure A1:**
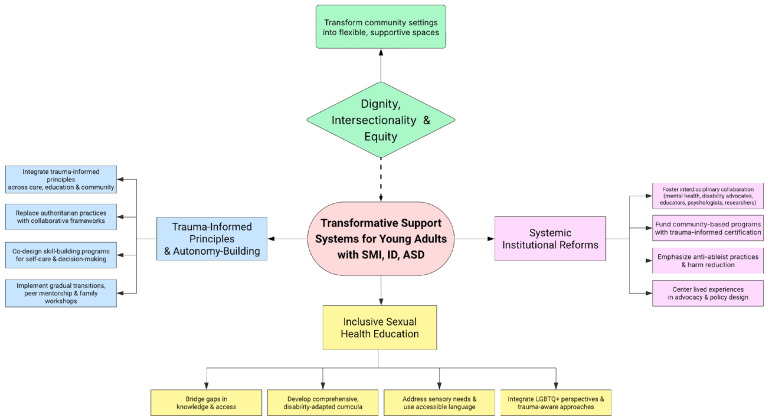
Visual representation of the implication for future supportive interventions. In the diagram, the dotted line represents a founding principle, which is a transversal value that influences the whole system, whilst the branches with solid lines represent operational, direct, and structural actions.

Ultimately, these interventions—rooted in dignity, intersectionality, and equity—aim to transform ecosystems into spaces where autonomy is nurtured, stigma is challenged, and systemic rigidity is replaced with flexibility. By bridging the divide between safety and empowerment, this study affirms the right of every individual to explore their identities, relationships, and aspirations on their own terms. A visual and schematic representation of these implications is provided in Appendix B (Figure A1) for further clarity. Future efforts must evaluate the scalability of these models across diverse contexts, ensuring that no one is left behind in the pursuit of holistic, rights-based care.

### 4.2. Limitation of This Study

This study’s findings, while insightful, must be interpreted within the context of its methodological constraints. The small sample size, though reflective of the challenges inherent in recruiting individuals with severe psychiatric and developmental conditions, limits the generalizability of results across the broader population of young adults with SMI, IDs, or ASD. The heterogeneity of diagnoses—spanning genetic syndromes (e.g., Down syndrome), psychotic disorders, and autism—introduces variability in psychosexual needs that complicates unified conclusions. Recruitment from a single community center may also introduce selection bias as participants were already embedded in a support system, potentially excluding those with limited access to care. Additionally, the reliance on self-reports and caregivers’ consent raises concerns about social desirability bias, particularly regarding sensitive topics like sexuality. The small sample size and diagnostic heterogeneity limit the reliability of sub-group comparisons as thematic saturation requires adequate representation within sub-groups, a condition not entirely met here. Future research with larger, balanced samples is needed to systematically explore the suggestions which have emerged from the comparisons. While the qualitative design provided rich, context-specific narratives, future research could strengthen validity through mixed-method approaches that triangulate subjective experiences with quantitative measures of psychosocial outcomes.

## 5. Conclusions

This study addressed three core research questions: (1) how young adults with SMI, IDs, and ASD negotiate bodily autonomy, gender identity, and sexuality; (2) the systemic barriers contributing to their psychosexual marginalization; and (3) how their lived experiences can inform rights-based interventions. The findings revealed pervasive infantilization, familial control over bodily autonomy, and fragmented sexual education, which collectively hinder participants’ psychosexual development. Societal stigma, gendered expectations, and institutional neglect emerged as key barriers, exacerbating vulnerabilities in relationships and self-perception.

Despite these challenges, participants demonstrated resilience and agency, advocating for inclusive interventions that prioritize autonomy and dignity. This study underscores the urgent need for trauma-informed, intersectional approaches—such as skill-building programs for independence and disability-adapted sexuality education—to address shared psychosexual needs across diagnostic groups. By centering lived experiences, this work challenges structural inequities and calls for systemic reforms to foster inclusive, rights-based care where psychosexual well-being is accessible to all. Future research should further explore the efficacy of such interventions in diverse community settings.

## Figures and Tables

**Table 1:** Socio-demographic characteristics of the participants.

Case	Range of Age	Type of Disability/ Diagnosis	Educational Qualification	Relationship Status	Living Status	First Intercourse	Number of Interviews
F1	between 25 and 30 years	Delusional disorder in the subject with an intellectual disability and obsessive-compulsive personality traits	High school diploma	In a romantic relationship	Parental unit	No	4
F2	between 25 and 30 years	Delusional disorder in the subject with an intellectual disability and obsessive-compulsive personality traits	High school diploma	In a romantic relationship	Parental unit	No	3
F3	between 25 and 30 years	Behavioral disorder in the subject with an average-grade intellectual disability	High school diploma	In a romantic relationship	Parental unit	No	3
F4	between 25 and 30 years	Medium–severe intellectual disability from birth asphyxia	High school diploma	Single	Parental unit	No	4
F5	between 31 and 40 years	Down syndrome	High school diploma	In a romantic relationship	Parental unit	No	4
F6	between 25 and 30 years	Medium–severe intellectual disability	High school diploma	In a romantic relationship	Parental unit	No	1
F7	between 25 and 30 years	Autism	High school diploma	Single	Parental unit	No	3
M1	between 25 and 30 years	Fragile X syndrome with psychotic symptoms	High school diploma	In a romantic relationship	Parental unit	No	3
M2	between 31 and 40 years	Williams syndrome with mild intellectual disability.	High school diploma	In a romantic relationship	Parental unit	No	1
M3	between 25 and 30 years	Down Syndrome	High school diploma	In a romantic relationship	Parental unit	No	1
M4	between 31 and 40 years	Down Syndrome	High school diploma	In a romantic relationship	Parental unit	No	2
M5	between 18 and 24 years	Schizophrenic-type graft psychosis	High school diploma	Single	Parental unit	No	4
M6	between 18 and 24 years.	Medium–severe intellectual disability.	High school diploma	In a romantic relationship	Parental unit	No	2

**Table 2:** Summary of emergent themes and sub-themes with key findings and relevant excerpts.

Theme	Sub-Themes	Key Findings	Relevant Excerpts
1. Relationship with the Body and Self-Image	Autonomy in Bodily Self-Care. Relationship with Body Image. Intimacy and Personal Boundaries. Psychosexual Development.	Participants demonstrated varied levels of independence in personal hygiene and self-care. Family influences (e.g., parental assistance) and social expectations (e.g., beauty standards) were significant in shaping self-image and the transition to sexual maturity. Experiences of blurred personal boundaries were noted, particularly in familial contexts, which also impacted psychosexual awareness.	I: So, at 18 you started to do everything yourself and before that who helped you with personal hygiene? M2: My mother. (M2, Pos 33) I: Why do you have short hair? F2: Because mom and dad make me cut it like a boy, since I was born. I: Why? F2: Because they don’t want long hair, that’s why! But my sister and I want long hair, not short! (F2, Pos 114–117). “When I started wearing makeup at seven, at my cousin’s friends’ house… my mom said, ‘No, don’t let my daughter wear makeup, because I’m afraid she won’t know how to handle lipstick, makeup, and she’s not used to it.’ Then, when she saw me older, she let me put some makeup on my eyes. But when I put on lipstick by myself, she got mad and said, ‘Who told you to put on lipstick now that you’ve grown up?’ And I said, ‘I did, Dad…,’ and Dad told me, ‘No, F1, wipe it off’” (F1, Pos 308). I: Does it happen that you go in while your brother showers?” F2: It happens, it happens. (F2, Pos 206–207)
2. Affectivity, Relationships, and Self-Development	Family Relationships. Romantic Relationships. Relationships with Educators/Secondary Caregivers. Emotionality and Self-Development.	Family dynamics ranged from supportive to conflictual, impacting emotional expression and autonomy. Romantic relationships were characterized by both idealization and real-life challenges, while interactions with educators often mirrored the authoritarian family model. Participants described emotional experiences (e.g., anger, fear) and their role in shaping self-concept and identity development.	F7 “Yes, I argue with mom […] I argued with her all the time.” (F4, Pos 371). “My passion is music... my grandfather got me into A. [Italian singer]” (M1, Pos 77) “Mom likes me and twin sister to dress the same, but then it’s bad because it’s like we are the same person. If we wear different clothes, we are two different people” (F2, Pos 388) “Love… when love happens, it’s always between two people who want each other… the spark ignites, and then comes the kiss” (M3, Pos 130). I: How do you get along with the babysitter? F2: With the babysitter? Terribly… she always bosses me around, just like the educators and my parents. (F2, Pos 228–229). “I’m an adult, but I also try to be like the kids… but I still try to be myself… I’m grown […] Being a man means being more… open.” (M3, Pos 36) I: “Do you think you are in a relationship or it is just friendship? M2: “I don’t know.” (M2, Pos 125–126).
3. Gender Identity and Sexual Orientation	Sexual Orientation and Attraction. Perception of Gender Identity. Social Roles and Gender Stereotypes. Engagement with LGBTQ+ Issues.	Participants articulated diverse sexual orientations and attractions, with some expressing rigid gender norms and others a more fluid understanding. Social expectations and stereotypes influenced their self-identification and behavior, while limited exposure to LGBTQ+ discourses affected their perceptions of gender roles.	I: ”(...) It depends, a girl can also choose to date girls, she doesn’t have to date compulsory boys. But what does F7 prefer?” F7: “I have to choose a boy, not a girl.” (F7, Pos 417–418). F1: LGBT means that all people who are trans must love, they have to love.” I: So, LGBT only counts for trans people? F1: Yes, just for trans people. LGBT is a community where there are all those trans people you see on the street who say you have to learn to love trans people. I heard it on ‘Catfish’. (F1, Pos 1002–1004). M1: I don’t like seeing a guy kiss another guy, it disgusts me. I: What bothers you? M1: A girl kissing another girl made me want to hit myself, to punch myself! It really upset me! (M1, Pos 400–401). F1: “For example, girls buy girls’ clothes, wear high heels, watch girls’ cartoons, and watch movies for girls, like ’The World of Patty’ and ’The Incorrigibles.’ And how do I know if you are a boy? The same way, you buy boys’ clothes, and then what do you do? You smoke, you watch boys’ cartoons...” (F1, Pos 931)
4. Sexuality	Sexual practices, fantasies, and behaviors. Intimacy, desire, and emotional tensions. Sexual education and cultural influences.	Participants had a fragmented and often inaccurate understanding of sexuality, mostly linking it to reproduction and marriage, with common misconceptions about contraception. Due to inadequate sexual education, they relied on sources like pornography, peers, and the media, leading to distorted perceptions of intimacy. Sexual curiosity was mixed with anxiety, fear, and shame, and unclear boundaries led some to reject certain acts due to discomfort or social stigma. Their romantic and sexual fantasies, shaped by fairy-tale narratives and the media, idealized love and marriage, contrasting with their limited real-world experiences. Families and professionals often avoided discussions, focusing on restrictions rather than education, while some institutional interventions were intrusive and counterproductive. Cultural norms reinforced heteronormative expectations, marginalizing LGBTQ+ identities and framing marriage strictly as a male–female union for procreation.	F1: Some people take ‘condoms’ so, they don’t have children while having intercourse. I: Do you know what a condom is? Have you ever seen one? F1: No, but I think it’s those pills, like medicine, that you take by mouth before having sex. (F1, Pos 871–873) I: What can boys and girls do together if they are in an intimate relation? F7: Love… they make love… I: And what does making love mean? F7: I don’t know, it’s talking, maybe… (F7, Pos 334–336) I: How do you feel when your boyfriend puts his hands in your breasts? F3: I feel bad. It is bad. (F3, Pos 750–751). F2: I heard about these three types of sexuality from my dad. Oral sex, physical and psychological. F2: Did he explain what they are? F2: No. (F2, Pos 669–671). I: And where did you learn? M3: I learned by myself. I: What did you use to learn? M3: I watched… because I watch videos… porn. I: When do you watch them? M3: On my phone. (M3, Pos 282–289) “There’s one thing I don’t like, when my therapist says, ‘touch yourself!’ I really hate it!” (M1, Pos 505). “As soon as [the educator] entered… he separated us because I’m a Down boy, and she’s a Down girl… two Down individuals can’t have sex...” (M3, Pos 247) I: What does making love mean? F2: Once you are married, you make love—when you want to have a child. (F2, Pos 506) M6: I used to see it too [pornography] on my cousin’s mobile phone. It is better not to see. I: No? M6: No, it’s disgusting. (M6, Pos 803–805).

## Data Availability

The data presented in this study are available on request from the corresponding author.

## Abbreviations

The following abbreviations are used in this manuscript:

ID	Intellectual Disability
ASD	Autism Spectrum Disorder
SMI	Severe/Serious Mental Illness
CSE	Comprehensive Sexuality Education

## Appendix A

**Table A1:** Interview questions.

Body perception	What is the difference between the body you had as a child and the one you have now? Which parts of your body do you consider most important to you? Why? How do you take care of your body? Do you wash and dress yourself? Do you look at yourself in the mirror? What do you think about your image?
Affectivity and emotional expression	Do you experience emotions? Which emotions do you feel most often and on what occasions do you experience them? In which parts of your body do you happen to feel the emotions you mentioned? What is friendship to you? What is the difference between being a friend or being in love? How is the relation with your family? How is the relation with the educators? How do you behave between friends?
Intimate relationships	What is love to you? How do you know if you are in love? What makes you fall in love? How do you know if the other person is in love with you or not? How to behave between lovers? Are you in love? Are you in an intimate relation? How do you behave with your partner?
Gender identity construction	How would you define yourself? A child, a teenager, an adult? How would you define yourself? A boy, a girl or something else? What is the difference between male and female? How can you say that you are a male/female/other?
Sexuality	Have you ever heard of sexuality? Where have you heard of it? What do you think sex is? What do you think making love is? How to have sex? Who can have sex? Why do people have sex? Do you know what pornography is? Have you ever heard of it?
